# Cell-targeted vaccines: implications for adaptive immunity

**DOI:** 10.3389/fimmu.2023.1221008

**Published:** 2023-08-16

**Authors:** Trevor Ung, Nakisha S. Rutledge, Adam M. Weiss, Aaron P. Esser-Kahn, Peter Deak

**Affiliations:** ^1^ Pritzker School of Molecular Engineering, University of Chicago, Chicago, IL, United States; ^2^ Chemical and Biological Engineering Department, Drexel University, Philadelphia, PA, United States

**Keywords:** vaccine, innate immunity, targeted vaccines, cell targeting, adaptive immunity

## Abstract

Recent advancements in immunology and chemistry have facilitated advancements in targeted vaccine technology. Targeting specific cell types, tissue locations, or receptors can allow for modulation of the adaptive immune response to vaccines. This review provides an overview of cellular targets of vaccines, suggests methods of targeting and downstream effects on immune responses, and summarizes general trends in the literature. Understanding the relationships between vaccine targets and subsequent adaptive immune responses is critical for effective vaccine design. This knowledge could facilitate design of more effective, disease-specialized vaccines.

## 1 Introduction

Vaccines are one of the most valuable human health technologies in terms of lives saved. From Edward Jenner’s variolation experiments with cowpox in the 18^th^ century, to Jonas Salk’s efforts to develop inactivated whole pathogen vaccines for polio, vaccine research has produced some of the most important medical breakthroughs in history. For a vaccine to stimulate an immune response against a specific pathogen, it must contain antigens related to that pathogen. First generation vaccines generally consist of live or attenuated/inactivated whole pathogens. Despite their historical success and widespread adoption, whole pathogen vaccines pose safety concerns as they contain extraneous and potentially harmful pathogen components. They may also replicate or revert to their pathogenic form (1). Subunit vaccines instead contain only the minimal components of the pathogen, such as a recombinant protein, required to stimulate an immune response. These technologies have improved the safety profile of vaccines (2). However, subunit vaccines are inherently less potent than whole pathogen vaccines at stimulating immune responses. Because of this limitation, they generally contain additional immunostimulatory molecules, known as adjuvants, to develop protective immunity (2). Other recent vaccine technologies include viral vector and nucleic acid-based vaccines, which encode for pathogenic antigens and induce antigen production in the cells of the vaccine recipient. In the case of viral vector vaccines, additional immune stimulation is provided through mimicry of natural infection, while in nucleic acid vaccines, innate immune stimulation is generated by components of the nucleic acid delivery system or by incorporation of additional adjuvants (3, 4). These technologies have been employed against pathogens including the Zika virus, and more recently, SARS-CoV-2 (5, 6).

A common feature of these emerging vaccine technologies is that they seek to improve and optimize immune responses through the choice of antigen or adjuvant, thereby tuning downstream adaptive immune responses. While the choice of antigen and adjuvant strongly influence the immune response, the type of cells that respond to, or are even targeted by the vaccine are critical to immune responses (7–9). This review will focus on this emerging role in vaccine research: the selective targeting of innate immune cells.

The immune system can be broadly separated into two categories: innate and adaptive immunity. Innate immune cells have evolved to recognize, bind, and capture pathogens. They play a crucial role in initiating the pathogen-specific immune response. Pathogen associated molecular patterns (PAMPs) are molecular motifs common to bacteria and viruses that bind to pattern recognition receptors (PRRs). PRRs are ubiquitously expressed on innate and other immune cell types (8). PAMP-PRR binding triggers inflammatory signaling, which typically results in pathogen phagocytosis, antigen presentation, and activation of innate immune cells (10). Vaccine adjuvants take advantage of this interaction, using PAMPs or mimicking their structure to mimic pathogen-associated immune activation in innate immune cells (11).

Innate immune cells that capture antigens and are activated by pathogens or adjuvants can be broadly classified as antigen-presenting cells (APCs). APCs serve as the bridge between innate and adaptive immune responses by processing antigens and presenting them to T cells. Presentation of pathogen-specific antigens to CD4^+^ “helper” T cells in the presence of appropriate innate immune signaling (e.g. PRR activation) initiates an adaptive immune response. APCs and CD4^+^ T cells further signal to naïve T cells and B cells, generating pathogen-specific CD8^+^ “killer” T cells as well as B cell maturation and neutralizing antibody responses. The initialization of adaptive immunity is a critical function of innate immune cells, making them attractive targets for vaccines (9).

**Figure 1:**
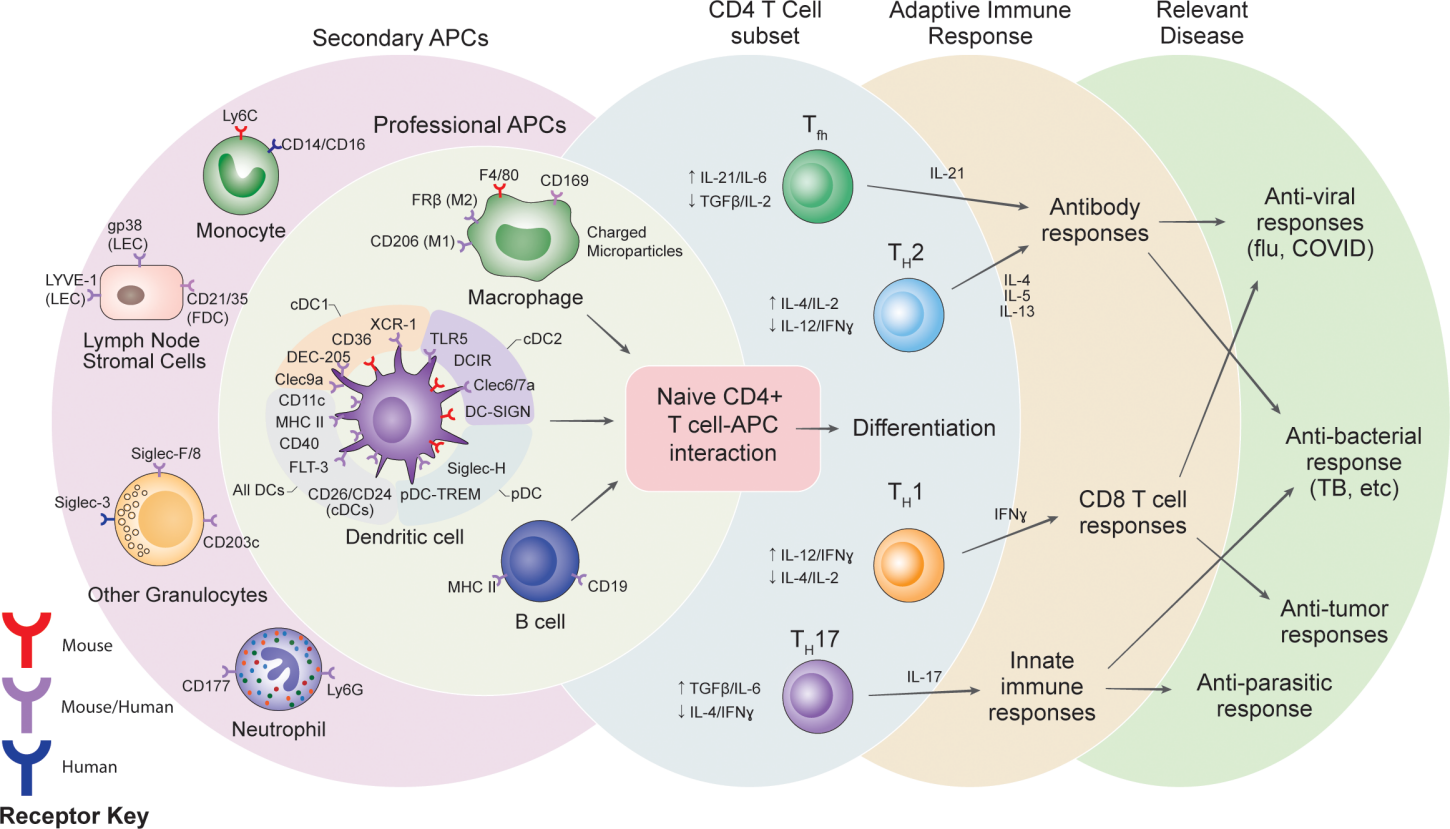
Summary of potential receptor targets for vaccines, the cells targeted and the potential variations in adaptive immune responses.

The vaccine-induced adaptive immune response can vary widely based on the specific spatiotemporal patterns of PRR activation and innate immune stimulation. Several reviews have extensively commented on how the choice of adjuvant and PRR stimulation modulates the type of adaptive immune response (12–14). Broadly, adaptive immune responses can be categorized based on the CD4^+^ T cell activity: conventional Th1, Th2, Th17 and T follicular helper (Tfh) cells, and regulatory T cell (Treg) subtypes (**Figure 1**) (15–17). Each response profile can be characterized by distinct cytokines released from APCs and T cells, which in turn, modulate downstream cellular and humoral responses. Generally, Th1 responses, characterized by IFN-γ and IL-12 signaling, generate CD8^+^ cytotoxic T cell responses and protect against intracellular pathogens (type 1 immunity). Th2 responses, characterized by IL-4, IL-5, and IL-13 signaling, generate antigen-specific antibodies, recruit granulocytes, and protect against parasites (type 2 immunity). Th17 responses, characterized by IL-17 signaling, generate inflammatory responses at mucosal membranes and protect against extracellular bacteria and fungi (type 3 immunity). Tfh cell responses, characterized by IL-21 signaling, assist and modulate the B cell response (15, 18, 19). Treg responses are characterized by secretion of immunosuppressive factors such as TGF-β, IL-10, and IL-35 (15, 20). Tregs limit immune responses towards antigens, such as self- and dietary antigens, to prevent autoimmunity and aberrant inflammatory responses, and also participate in the resolution of immune responses to pathogens (20). While Tregs can also be induced alongside conventional T cells during vaccination (21), the focus of this review is on the conventional T cell responses that are required for adaptive immunity towards pathogens. Specific responses are desired against different diseases – for example, in viral infections, a Th1 response is more favorable (9, 11, 22). Thus, there is great interest in tuning T cell responses during vaccination. While refining adjuvant formulations can modulate cytokine production to exert a degree of control over CD4^+^ T cell responses, this is still an emerging field of research (23). An additional and underutilized “lever” for controlling T cell responses to vaccines is modulating which innate immune cells act as APCs, capturing and presenting antigen to T cells (9, 24, 25). Here we will discuss the types of innate immune cells that have been targeted, the targeting systems employed, and their downstream effect on adaptive immune responses (see **Table S1** for comprehensive list of all targeting strategies referenced).

## 2 Innate immune cell subsets

The innate immune system can be broadly categorized into compartments based on their primary function: leukocyte killer cells, granulocytes, and phagocytic cells. Leukocyte killer cells, such as natural killer cells (NK cells), directly attack and lyse infected host cells, and are generally involved in viral infections and cancer (26, 27). Granulocytes such as neutrophils, basophils, mast cells, and eosinophils, contain large depots of cytotoxic molecules in granules that can be released into circulation upon activation, and are generally involved in parasitic and allergic responses (28). Phagocytic cells such as macrophages, monocytes, and dendritic cells, phagocytose pathogens and present them to naïve T cells to initialize an adaptive immune response (29). While each subset may be involved in vaccine-induced immune responses, phagocytic cells are the primary focus of this review, as these are the primary APCs involved in T cell responses. Furthermore, phagocytic cells can also be classified into further subsets, which are discussed below.

### 2.1 Dendritic cells

Dendritic cells (DCs) present the most attractive target for vaccines due to their increased capabilities for antigen presentation, lymph node migration, and T cell stimulation, compared to other APCs (30, 31). Dendritic cells are canonically identified by their expression of the integrin CD11c and major histocompatibility complex class II (MHC II), as well as characteristic dendrite projections that appear when the cells are fully matured (30). DCs can be broadly classified based on their cell origin: conventional DCs (cDCs) arise from myeloid progenitor cells, plasmacytoid DCs (pDCs) have been reported to arise from both myeloid and lymphoid progenitors, and monocyte-derived DCs (moDCs) arise from monocytes after inflammatory stimulus (32–39). Additionally, human pDCs have limited expression of CD11c (31, 34, 40–42). These subsets will be discussed specifically later in this review. In this section, we will only discuss strategies that broadly target all DCs.

Receptors that have been used for pan-DC targeting include CD11c, MHC II, CD40, GM-CSFR (CD116), and FLT3 (CD135) (40, 43). Here, “pan-DC” indicates a receptor has been used to target all DCs, but not necessarily for DC-specific targeting. Some of these receptors are expressed on other immune cells and are thus not DC-specific (e.g. macrophages may express CD11c and MHC II while B cells also express MHC II and CD40) (43, 44). However, given the increased capacity of DCs for antigen presentation compared to other APCs (30, 31), vaccines targeting these markers have attributed the immunological outcomes to DC targeting. Several vaccines have targeted pan-DC markers to improve T cell responses. A CD11c-targeting DNA vaccine, which produces anti-CD11c single chain variable fragments (scFvs) fused to tumor-associated antigens, produced strong anti-tumor CD8^+^ T cell responses in mice (45). Similarly, liposomes bearing anti-CD11c scFvs, and containing the model antigen ovalbumin (OVA) and either lipopolysaccharide (LPS) or IFN-γ as adjuvant, improved anti-tumor CD8^+^ T cell responses and slowed progression of OVA-bearing tumors (46). CD11c can also be targeted using peptide ligands. A peptide derived from ICAM-4, a natural ligand for CD11c, has been used to target nanocarriers to DCs, but in the context of delivering tolerogenic compounds (47, 48). Other peptide ligands for pan-DC targeting can be generated by phage display, although the cell-specific targets may be unknown. Liu et al. generated DC-targeting peptides using phage display against splenic DCs (49). The resultant peptides were used to target silica-based nanoparticles containing OVA and cytosine phosphoguanine (CpG) to DCs, improving CD8^+^ T cell responses against OVA-bearing tumors (49). Puth et al. used a similar phage display strategy against CD11c^+^ cells (50). The resultant peptides were used to target a recombinant protein, consisting of tumor antigens and flagellin as an adjuvant, to DCs. This elicited strong anti-tumor T cell responses and slowed tumor progression (50). This indicates targeting antigen to DCs through CD11c can effectively produce antigen-specific CD8^+^ T cells.

Cruz et al. compared immune responses between DC targeting with PLGA nanoparticles using two pan-DC markers, CD11c and CD40 (51). Anti-CD11c or anti-CD40 monoclonal antibodies (mAb) were adsorbed on PLGA nanoparticles containing OVA and two adjuvants, polyinosinic:polycytidylic acid (poly I:C), and resiquimod (R848). Both CD40- and CD11c-targeting nanoparticles improved CD8^+^ T cell cytokine production and cytolytic activity to similar degrees, compared to non-targeting nanoparticles (51). However, Castro et al. compared the T cell responses after vaccination with OVA directly conjugated to anti-CD11c or anti-MHC II antigen-binding fragments (Fabs) (52). With Fab-antigen conjugates, CD11c targeting elicited better CD8^+^ and CD4^+^ T cell responses compared to MHC II targeting (52). Taken together, this suggests that the magnitude of the T cell response can depend on the choice of pan-DC marker, but that the targeting method can reinforce or negate any differences observed by targeting one marker over the other.

Targeting pan-DC markers can also benefit antibody responses. Wang et al. immunized mice with hamster anti-mouse CD11c and goat anti-hamster secondary antibodies, generating immune complexes targeting CD11c (53). This strategy rapidly increased anti-goat IgG levels over time and improved anti-goat serum titers compared to an isotype control. A limitation of this study is that T cell responses were not tracked (53). Additionally, the formation of immune complexes is known to trigger phagocytosis and innate immune cell activation through the complement system, which is known to trigger antibody responses (54). Pugholm et al. showed that immunizing mice with rat anti-mouse CD11c antibodies alone increased anti-rat IgG production (55). This suggests CD11c targeting alone, even in the absence of immune complex formation, can contribute to an increase in antibody levels. Braathen et al. generated DNA vaccines which produced hemagglutinin (HA) from influenza fused to scFvs against CD11c, CD40, and MHC II (56). Targeting CD11c and MHC II produced the highest magnitude of IgG of all the pan-DC markers. A study from White et al. similarly showed that targeting *via* anti-CD11c or anti-CD40 Fabs markedly raised antibody titers compared to MHC II Fabs (57). Overall, targeting pan-DC markers increased overall antibody titers, and CD11c seems to elicit the highest magnitude of IgG. These studies did not assess T cell functionality, nor the epitope diversity or affinity of these antibodies. However, the choice of pan-DC marker could influence the IgG subclass produced. Braathen et al. showed that CD11c and MHC II targeting produced equal amounts of IgG1 and IgG2, while GM-CSFR targeting resulted in an IgG1-biased response, and CD40 or FLT3 targeting resulted in an IgG2-biased response (56). Since IgG1 and IgG2 are associated with Th2 and Th1 responses, respectively (58), this suggests that the choice of pan-DC targeting marker can influence the type of adaptive response.

DCs may also be broadly targeted *via* scavenger receptors, which recognize a broad range of ligands including both endogenous and pathogenic molecules, and are thus involved in homeostasis and immune responses (59). The scavenger receptor, CD206 (also known as mannose receptor (MR)), is a C-type lectin receptor (CLR) that recognizes pathogen glycoproteins, and is expressed on immature DCs (60–63). Wilson et al. designed a targeted glyco-adjuvant consisting of a random copolymer of mannose and TLR7 agonist (64). When conjugated to an antigen, the mannose allows delivery to DCs *via* CD206 and activation *via* TLR7. The glyco-adjuvant improved the magnitude of the antigen-specific CD8^+^ and CD4^+^ T cell response, as well as the magnitude and epitope coverage of the IgG response. CD206 has also been targeted *via* antigens conjugated to sulfated glycans, and Singh et al. showed that such targeting was able to induce both CD8^+^ T cell responses and a Th1-skewed CD4^+^ T cell response (65). He et al. showed that targeting of humanized CD206 with antigen-mAb conjugates in a transgenic mouse model also improved both CD8^+^ and CD4^+^ T cell responses, demonstrating the ability to target human CD206 (66). However, in humans CD206 has only been observed on moDCs, limiting its use for human pan-DC targeting (67). Additionally, CD206 and other scavenger receptors that recognize glycosylated proteins are widely expressed on other innate immune cell subsets, including macrophages (68). While CD206 targeting may broadly benefit adaptive immune responses, the lack of selective targeting makes it difficult to attribute these outcomes to DCs.

Overall, targeting of pan-DC markers generally improves both cellular and humoral immunity in vaccination. The choice of targeting marker remains an understudied method by which specific adaptive immune responses may be generated. At present, it seems the choice of pan-DC target and its effect on adaptive immune bias are overshadowed by the model antigen studied, the delivery vehicle, and the presence, absence, or type of adjuvant. Furthermore, lack of specificity of the receptor should be considered. Receptors such as CD11c, CD206, CD40 and MHC II are also expressed on other innate immune subsets, such as macrophages and B cells, and thus off-target or additive effects of vaccines targeting these receptors should be studied.

#### 2.1.1 Conventional dendritic cells

Conventional dendritic cells (cDCs) are the most common DC subset (43, 69). cDCs can be identified based on their location: lymphoid resident cDCs sample antigens through draining lymph, while peripheral cDCs traffic to lymph nodes after capturing antigen. Within lymphoid tissues, such as lymph nodes, they can then present antigen to and activate naïve T cells. As with all DCs, cDCs commonly express CD11c and MHC II, but these markers also arise in pDCs, and on macrophages at lower levels (43).

CD24 and CD26 are potential pan-cDC surface targets, but are also present on a wide variety of immune and stromal cell types (31, 70–75). Antibody targeting of these receptors have been used in contexts outside vaccination, and could potentially enable a platform to target cDCs (75–77). However, given their expression in other tissues, they may not provide selective targeting of cDCs.

Other cDC markers are used to delineate the two subsets of cDCs, conventional type 1 and type 2 DCs (cDC1s and cDC2s). There are two important clarifications for cDC subtypes. First, while expression of transcription factors and surface molecules is a widely accepted method of classification, recent research has shown heterogeneity in these populations, especially during active infection (78). Furthermore, recent single cell sequencing studies suggest other categories of cDCs, particularly in humans, with nuanced lineages and functions (79, 80). As we are focusing on receptor targeting and consequences on downstream effector responses, rather than the intricacies of innate immune classification, here we will rely on established cDC1 and cDC2 markers and briefly overlook newer paradigms of classification for simplicity.

With regard to vaccine design; cDC1s and cDC2s function differently when activating T cells, making them potential targets for vaccines which can modulate T cell responses (43). In mice, cDC1s are generally associated with type 1 immune responses, including cross-presentation of exogenous or cell-associated antigen on major histocompatibility complex class I (MHC I). As a result, cDC1s primarily facilitate CD8^+^ T cell responses and release Th1-biasing cytokines (33, 41, 81, 82). cDC2s are generally associated with type 2 and type 3 immune responses, including capturing soluble antigen. As a result, cDC2s primarily facilitate CD4^+^ T cell and antibody responses (33, 41, 81–84). However these delineations are less strict in human cDCs, and under certain conditions human cDC1s can facilitate type 2 immune responses, while conversely human cDC2s can facilitate CD8^+^ T cell and type 1 immune responses (41, 85, 86). The next sections will discuss cDC1 and cDC2 targeting in detail.

##### 2.1.1.1 Conventional type 1 dendritic cells

cDC1s are typically identified by their expression of CD8α in mice, and CD141 (BDCA3) in humans (31, 33, 41, 82, 87). cDC1 targeted vaccines are often intended to invoke type 1 immune responses, particularly for the generation of antiviral or anti-tumor immunity (51, 88). Receptors that have been used for cDC1 targeting include XCR1, DEC-205 (CD205), Clec9a (DNGR1), Clec12a, Treml4, CD36, LOX1, and FcγR (51, 52, 55, 56, 62, 88–105).

XCR1 is a receptor for the chemokine XCL1, which plays a role in cDC1 recruitment (106). XCR1 has been targeted by DNA vaccines encoding XCL1-antigen fusion proteins. It has been shown to generate strong CD8^+^ T cell responses, as well as Th1-biased CD4^+^ T cell and IgG2 antibody responses against OVA and viral antigens (56, 93, 99, 100, 107). DEC-205, Clec9a, and Clec12a are CLRs, which similar to CD206, broadly participate in pathogen recognition, endocytosis, and antigen uptake (95). T cell and antibody responses to CLR targeting are strongly dependent on the inflammatory context. Recombinant anti-DEC-205 and anti-Clec12a mAb-antigen conjugates have been shown to raise strong antigen-specific CD8^+^ T cell responses in the presence of adjuvant or inflammation (88, 93, 95, 97, 98). DEC-205 targeting in human DCs also generates both antigen-specific CD8^+^ and CD4^+^ T cell responses (108–110). Meixlsperger et al. showed that this response required maturation of CD141^+^ DCs (109), a marker associated with cDC1s in humans. However, under steady state or low-inflammatory conditions, targeting DEC-205 and Clec12a raises weak or even tolerogenic responses (93–95, 97). Additionally, DEC-205 and Clec12a are not entirely cDC1 specific. DEC-205 is also expressed at low levels on cDC2s in mice (93), but it is broadly expressed in DCs in humans (62, 96). Clec12a, meanwhile, is also expressed on pDCs in mice but broadly on DCs in humans (97). Thus, interpretation of the immune responses resulting from DEC-205 or Clec12a targeting can be confounded by adjuvant effects and differential specificity of these receptors for cDC1s between species.

The last CLR of interest for cDC1, Clec9a, has a high specificity for both mouse and human cDC1s (111, 112). Clec9a naturally binds F-actin-myosin complexes associated with dying cells, and its activation is associated with phagosomal rupture and cross-presentation of antigens to CD8^+^ T cells (113). Studies comparing Clec9a and DEC-205 targeting in mice *via* mAb-antigen conjugates showed that despite raising similar CD8^+^ T cell responses, Clec9a targeting induced better protection in an influenza challenge model (93). In addition, Clec9a targeting elicited antibody and Tfh responses in both mice and non-human primates (114, 115). However, there are conflicting studies on whether or not adjuvant is required to produce Tfh and antibody responses resulting from Clec9a targeting (95, 115–117). In human DCs, Clec9a targeting was highly specific for CD141^+^ DCs (96). Additionally, when Clec9a was targeted with tumor antigens and poly I:C as adjuvant, anti-tumor CD8^+^ T cell responses were stronger than DEC-205 targeting (96). Taken together, Clec9a targeting of cDC1s has the potential to improve both cellular and humoral responses in vaccination, like targeting the pan-DC receptor CD11c.

Several other non-CLR scavenger receptors have been targeted on cDC1s. TREML4 is a receptor that mediates uptake of apoptotic or necrotic cells, and is primarily associated with cDC1s (89). Anti-TREML4 mAb-antigen conjugates can generate antigen-specific CD8^+^ and CD4^+^ T cell immunity to tumor and viral antigens (90), or antigen-specific tolerance (91), when targeted in the presence or absence of external adjuvant. CD36 is a scavenger receptor that has high specificity for mouse CD8α^+^ DCs (98). In a comparative study between anti-CD36 and anti-DEC-205 scFv-antigen conjugates, CD36 targeting was able to induce CD8^+^ and CD4^+^ T cell responses, even in the absence of adjuvant (98). LOX1 is another scavenger receptor that recognizes heat shock proteins (Hsp), is involved in cross presentation, and has high specificity for CD8α^+^ DCs (102–104, 118). LOX1 targeting *via* antigen fused to anti-LOX1 mAbs or Hsp-60 induced strong CD8^+^ T cell responses, and to a lesser extent, CD4^+^ T cell responses (102–104, 118). Altogether, while these studies show that TREML4, CD36, and LOX1 are potential cDC1 targets and can raise both CD8^+^ and CD4^+^ T cell responses after vaccination, bias towards Th1 or Th2 responses generated by activation of these receptors is unclear or understudied. Comparing vaccines targeting these receptors to other targets such as XCR1, with a more well-defined Th1 bias, could help evaluate if targeting these receptors can induce a bias towards type 1 or type 2 immune responses.

Another family of receptors that have been used to target cDC1s is the Fc-gamma receptor (FcγR). It is important to consider the differences between murine and human FcγRs, as well as the fact that FcγR isoforms have activating or inhibitory downstream signaling (119–122). Activating isoforms contain immunoreceptor tyrosine-based activation motifs (ITAMs) while inhibitory isoforms contain immunoreceptor tyrosine-based inhibition motifs (ITIMs) (121, 122). Murine DCs express the activating isoforms FcγRI, FcγRIII, FcγRIV, and the inhibitory isoform FcγRIIb, while human DCs express the activating isoforms FcγRI, FcγRIIa/c, and the inhibitory isoform FcγRIIb (119, 121). FcγRIV is not found in human DCs, while FcγRIIa/c are not found in murine DCs (121). These species-specific isoforms are preferentially expressed by cDCs both in mice and humans (119). Lehmann et al. showed that mAb-antigen conjugates targeting FcγRIIb, FcγRIII, and FcγRIV on murine DCs were preferentially taken up by CD8α^+^ DCs compared to CD8α^-^ DCs, suggesting some cDC1 targeting ability (105). However, the shared expression of these isoforms with cDC2s and pDCs may limit cDC1-specific targeting, and FcγRIIb has also been used to target murine pDCs (which will be discussed in a later section). Lehmann et al. observed CD8^+^ and CD4^+^ T cell responses when targeting both FcγRIV and FcγRIIb (105). However, long-lasting T cell responses were dependent on the presence of adjuvant rather than the isoform targeted, suggesting targeting of activating or inhibitory isoforms alone has minimal effect. Additionally, CD8^+^ T cell responses were only induced by CD8α^+^ DCs while CD4^+^ T cell responses were induced primarily by CD8α^-^ DCs. Similarly, Flinsenberg et al. used IgG-coupled antigen to broadly target FcγR on human DCs, and showed that while cDC2s took up more antigen, cDC1s generated a stronger CD8^+^ T cell response, likely due to the increased capability of cDC1s for cross-presentation (101). While this suggests FcγR targeting can improve CD8^+^ T cell responses, the lack of FcγR specificity for cDC1s should be considered, as well as any adjuvant effects.

cDC1s have often been targeted for their cross-presenting capabilities and to raise type 1 immune responses. Receptors with clear specificity for cDC1s, such as XCR1, have been associated with strong effector T cell response against viral antigens and tumors (123). Alternatively, markers with less clear selectivity for cDC1 show increased type 1 immune responses, but less clear type 1 bias, which may be a result of the lack of receptor selectivity. This hypothesis is further supported by the fact that cDC1s show strong MHC I and cross-presentation of exogenous antigen, facilitating formation of type 1 responses (124). Yu et al. have reported that human lung cDC1s are able to generate type 2 immune responses (85). However, this may be tissue-dependent, as the responses were dependent on induction of OX40L on cDC1s by the epithelial cytokine TSLP (85). Overall, it is likely that targeting cDC1 takes advantage of this cross-presentation and therefore is more suitable for generating anti-viral and anti-tumoral type 1 skewed immune responses.

##### 2.1.1.2 Conventional type 2 dendritic cells

cDC2s are typically identified by expression of CD11b and lack of CD8α in mice, or by CD1c (BDCA1) in humans, while CD172a (SIRPα) is used as a marker for both humans and mice (31, 33, 41, 82, 87). Receptors that have been used for cDC2 targeting include DCIR (Clec4a), GM-CSFR (CD116), CCR1/3/5, TLR5, Dectin-1 (Clec7a), Dectin-2 (Clec6a), DC-SIGN (CIRE/CD209), FIRE, and MGL (Clec10a/CD301) (55, 56, 91, 125–134).

While there are 4 murine isoforms of the CLR DCIR (DCIR1-4), the isoform DCIR2 is found specifically on CD8α^-^ cDCs and is the most specific receptor for mouse cDC2s (91, 125–128, 130, 135–138). Anti-DCIR2 mAb-conjugates preferentially induced antigen presentation on MHC II, CD4^+^ T cell activation, and antibody generation, particularly in comparison to cDC1-targeting anti-DEC-205 mAb-conjugates (125, 126, 128, 130). However, these responses were highly dependent on the presence of an adjuvant or inflammatory stimulus (125, 126, 128). Surprisingly, DCIR2 targeting induced both CD8^+^ T cell responses and IgG2 production, and induced equal protection in a tumor challenge model compared to DEC-205 targeting (128). This suggests that, while cDC2s are generally associated with CD4^+^ T cell and antibody responses, they can also participate in type 1 immune responses. Humans express a single DCIR isoform with closest homology to murine DCIR1 and DCIR2 (136, 137). Klechevsky et al. showed that targeting of DCIR *via* mAb-antigen conjugates efficiently induced cross-presentation in human DCs and generation of CD8^+^ T cell responses, with and without adjuvant (139). However, DCIR is broadly present on human DC subtypes including pDCs (136–139), limiting the ability of DCIR to be considered a cDC2-specific target in humans. An additional consideration for vaccine targeting *via* DCIR is that both mouse and human DCIR contain ITIM domains, and targeting DCIR in the absence of adjuvant has been used to elicit tolerogenic responses (137, 140, 141). Despite this, the work done by Lehmann et al. targeting activating and inhibitory isoforms of FcγR and the work by Klechevsky et al. targeting DCIR altogether suggest that targeting ITIM-containing receptors will not necessarily lead to inhibition of immune responses, but instead may depend on whether or not targeting occurs in an inflammatory or steady-state context (105, 139).

Braathen et al. and Lysén et al. compared the effect of cDC2 targeting through DNA vaccines expressing recombinant proteins with antigen and GM-CSF, CCL3-HA, or flagellin (56, 129). GM-CSFR is a receptor for GM-CSF, a cytokine which participates in DC differentiation (142, 143). CCL3 is a ligand for CCR1/3/5, which are expressed widely on DCs, including cDC2s (129). TLR5 is a receptor that recognizes flagellin, which is preferentially expressed on CD11b^+^ DCs compared to other APCs (25, 144, 145). In a standard subcutaneous or intramuscular vaccination, it could be argued that the flagellin fusion proteins preferentially target cDC2s given the limited TLR5 expression in major APC subsets that reside in these tissues (144). Likewise, while GM-CSFR and CCR1/3/5 are also not specific to cDC2s, fusion proteins targeting antigens to these receptors preferentially bound cDC2s for all constructs, suggesting some cDC2 targeting ability (56, 129). More interesting is the downstream effect of GM-CSFR and CCR1/3/5 targeting; each fusion protein influenced the IgG subclass, with GM-CSF producing an IgG1 bias, CCL3 producing an IgG2 bias, and flagellin producing equal IgG1 and IgG2 (56). While it is tempting to conclude that both type 1 and type 2 immune responses can be generated by targeting cDC2s expressing these receptors, the lack of specificity for cDC2 should be considered, as both TLR5 and CCR1/3/5 are upregulated in, but not limited to, cDC2s.

Dectin-1 and Dectin-2 are CLRs which are commonly associated with CD8α^-^ and CD1c^+^ DCs, suggesting cDC2 expression in mice and humans (41, 131, 132, 146). In mice, subcutaneous vaccinations where Dectin-1 or Dectin-2 mAbs were conjugated to antigens and formulated with poly I:C as adjuvant resulted in equal CD8^+^ and CD4^+^ T cell responses (131, 132). Meanwhile, when Dectin-1 was targeted *via* intravenous vaccination, a CD4^+^ T cell response was generated instead (131). Carter et al. hypothesized that the balanced CD8^+^ and CD4^+^ T cell response was due to Langerhans cells, APCs that reside in the skin which express both Dectin-1 and DEC-205 (146). They suggested that during subcutaneous vaccination, Langerhans cells captured and presented antigen to both CD8^+^ and CD4^+^ T cells, while during intravenous administration, Dectin-1 expressing cDC2s preferentially captured and presented antigen to CD4^+^ T cells (131). In humans, Lundberg et al. reported a larger proportion of CD141^+^ DCs expressing Dectin-1 and Dectin-2 compared to CD1c^+^ DCs, suggesting Dectin-1 and Dectin-2 may be more highly expressed on human cDC1s (147). A caveat of this study is that the DCs studied were isolated from peripheral blood of mostly allergic patients, although expression patterns of Dectin-1 and Dectin-2 did not vary significantly between allergic and healthy patients (147). Additionally, while this study was performed on human DCs, the work by Lundberg et al. and Carter et al. highlight the difficulty of identifying cDC2-specific receptors in humans and mice, and subsequent interpretation of the effects on downstream immune responses. Pugholm et al. showed that targeting of Dectin-1 and Dectin-2 could also raise antibody responses in the absence of adjuvant (55). These results are consistent with pan-DC targeting strategies that raised antibody responses, as well as the ability of adjuvants to raise robust humoral and cellular immunity.

DC-SIGN is another CLR commonly found on CD8α^-^ and CD11b^+^ DCs in mice, suggesting preferential expression on cDC2s (133, 134). Corbett et al. immunized mice with rat anti-mouse DC-SIGN (mDC-SIGN) antibodies and showed significantly increased anti-rat IgG even in the absence of adjuvant (133). Schetters et al. further showed that targeting mDC-SIGN *via* mAb-antigen conjugates in the presence of adjuvant could also generate CD8^+^ and CD4^+^ T cell responses, consistent with previous studies targeting Dectin-1 and Dectin-2 (134). The targeted portion of the mDC-SIGN receptor may also play a role in determining T cell responses, as Tacken et al. showed that antibodies targeting the neck region of mDC-SIGN, rather than the carbohydrate recognition domain which is common to all CLRs, improved DC cross-presentation (148). The putative human analog of mDC-SIGN, hDC-SIGN, has only been observed on moDCs but not human peripheral DCs (67, 134, 149), suggesting hDC-SIGN may not be useful for cDC2-specific targeting in humans. FIRE (F4/80-like receptor) is a surface glycoprotein that is found on CD8α^-^ DCs in mice, but its functions are not currently known (150). Corbett et al. showed that similar to mDC-SIGN, immunizing mice with rat anti-FIRE antibodies significantly raised anti-rat IgG in the absence of adjuvant (133), indicating similar downstream functions as DC-SIGN targeting; however further research is required to fully investigate these claims.

MGL (macrophage galactose-type lectin) is an additional CLR found on DCs and macrophages in both mice and humans, which is preferential to cDC2 (151–154). Two isoforms exist in mice; the isoform MGL1 (CD301a) is found on macrophages while the isoform MGL2 (CD301b) is found on CD8α^-^ cDCs (153–155). Humans express a single isoform of MGL that is most homologous to MGL2, and is found primarily on CD1c^+^ DCs, suggesting MGL is a potential target for cDC2s (153, 155). Although to date, no studies have targeted MGL in vaccination, Heger et al. and Eggink et al. generated targeting peptides for MGL based off the endogenous ligand MUC-1, or identified *via* phage display (153, 155). Heger et al. showed that a glycosylated MUC-1 peptide preferentially bound human cDC2s *in vitro* (153). Heger et al. also noted that administration of the MUC-1 peptide enhanced DC cytokine secretion in combination with the adjuvant R848, consistent with previous reports that ligation of MGL augments TLR-induced DC cytokine secretion (151, 153). While Eggink et al. did not assess the targeting ability of their MGL-targeting peptides, they similarly showed that MGL-targeting peptides induced DC maturation and cytokine secretion in human DCs and modestly improved survival in tumor-bearing mice (155). Together, these studies suggest that not only is MGL a potential cDC2-specific target, but that targeting of MGL alone or in the presence of adjuvant can benefit vaccine responses and warrants further investigation.

Vaccines targeting cDC2s generate a breadth of responses, in contrast to cDC1 targeting. Depending on the targeted receptor, type 1- or type 2-skewed immune responses have been observed. In some cases, even tolerogenic responses have been observed, when cDC2s were targeted *via* DCIR2 in the absence of adjuvant (140, 141). Additionally, Bourdely et al. showed that human CD1c^+^ DCs could prime CD8^+^ T cells. CD1c normally marks cDC2s in humans, suggesting that cDC2s can also raise type 1 immune responses. However, these cells also expressed CD163 and were proposed to be a new subset of DCs, as they also expressed monocyte markers (86). Indeed, a complicating factor is that the markers for cDC2 are much more widely expressed on other innate immune cells, limiting the selectivity of cDC2 targeting. Therefore, it is unknown if the variety of responses are due to poor receptor selectivity, or intrinsic to cDC2 targeting. With that said, the data provided herein provide compelling evidence that targeting cDC2-related receptors can generate enhanced antibody and type 2 immune responses compared to cDC1 targeting.

#### 2.1.2 Plasmacytoid dendritic cells

pDCs are a small subset of DCs that circulate through the blood and are specialized for viral recognition and response. They express TLRs which recognize foreign nucleic acid, such as TLR7 and TLR9, and rapidly secrete type I and type III interferons (IFNs) in response to infection (31, 40, 156, 157). pDCs are typically identified by expression of B220 (CD45R), Ly6C, Siglec-H, and BST2 (CD317) in mice, BDCA2 (CD303), BDCA4 (CD304), and CD123 (IL-3R) in humans, and CD45RA in both (31, 40). Receptors that have been used for pDC targeting include PDC-TREM, Siglec-H, BST2, DEC-205, DCIR, BDCA2, and FcγRII (55, 158–164).

The main pDC receptors studied for targeted vaccination are PDC-TREM, Siglec-H, and BST2. PDC-TREM is a pDC-specific receptor that triggers type I IFN production (165). Siglec-H is a pDC-specific receptor that functions as an endocytic receptor, but unlike other receptors in the Siglec family, it does not bind sialic acids (162). Pugholm et al. showed that immunization with rat anti-PDC-TREM without adjuvant was able to raise anti-rat IgG, while anti-Siglec-H was unable to raise an antibody response (55). Targeting of Siglec-H by mAb-antigen conjugates without adjuvant led to a tolerogenic CD4^+^ T cell response (163), but in the presence of adjuvant a CD8^+^ T cell response was generated (162). BST2 is a receptor that prevents virion dissemination by binding virion lipid membranes (166). BST2 identifies pDCs under homeostatic conditions, but is upregulated on most cells during viral infection and inflammation (167). Similarly, targeting of BST2 by mAb-antigen conjugates in the presence of adjuvant generated a CD8^+^ T cell response and IgG2 production, suggesting a Th1 response (164). However, CD8^+^ T cell responses from pDC targeting is thought to occur through antigen transfer to cDC1s and not directly from pDC-T cell interactions (168). Taken together, this suggests that while PDC-TREM targeting can raise antibody responses without adjuvant, Siglec-H and BST2 targeting require adjuvant and potentially other DC subsets to generate humoral and T cell responses.

Due to their availability in blood, pDCs are more extensively studied in humans than other DC subsets. Human pDCs have been targeted *via* DEC-205, DCIR, BDCA-2, and FcγRII (158–161). Tel et al. demonstrated that when PLGA nanoparticles containing OVA and R848 were targeted to pDCs *via* these receptors, CD8^+^ and CD4^+^ T cell responses were generated (158). Human pDC-generated immune responses are likely dependent on the presence of adjuvant, as targeting and ligation of DCIR and BDCA2 alone can suppress DC activation (159, 160). To this end, Sepulveda-Toepfer et al. generated an αFcγRII-CpG conjugate that was shown to target and activate human pDCs (161); however, its efficacy in a vaccine formulation has yet to be assessed. Additionally, the presence of FcγRII on other immune cells such as cDC1s and its previous use as a cDC1 target, as well as the activating and inhibitory isoforms of this receptor, should be considered (101, 119–122). Overall, the literature indicates that targeting pDCs can generate type 1 immune responses, but the response can vary depending on the targeted receptor and on the presence of adjuvant. More research is required to fully establish the effects of pDC targeting during vaccination.

#### 2.1.3 Other DC subsets

Formulations administered *via* subcutaneous and intramuscular injection routes will encounter other types of cDCs that should be considered in the design of effective vaccines. Under steady state conditions, cDCs can be further classified into tissue resident and migratory subsets. This classification is determined on if they primarily reside in local tissue environments or are primed to sample antigens from local tissues and migrate to nearby lymphoid tissues (69). DC phenotypes can further vary by tissue location, such as lung and intestinal DCs. The classification and functions of these tissue-resident DCs have been reviewed extensively elsewhere (31, 73, 87, 142, 169–172); this review is focused on APCs in lymphoid tissues. In lymphoid tissues, migratory cDCs express more DC maturation markers, and have higher levels of MHC II and lower levels of CD11c compared to resident cDCs under homeostatic conditions (31). However, under inflammatory conditions, where both resident and migratory DCs will mature, this classification no longer holds (31). Instead, lymphoid resident and migratory cDC1s can be identified by expression of CD8α or CD103, respectively (31, 87). Semmrich et al. showed that targeting CD103^+^ DCs *via* mAb-antigen conjugates raised both CD8^+^ and CD4^+^ T cell responses in the presence of adjuvant (173). In the absence of adjuvant, a tolerogenic response resulted (173). CD103 is an integrin that plays a role in tissue residence for a wide variety of cell types, including T cells (174), and thus is a non-specific target for migratory cDC1s. However, the immunogenic response is consistent with the importance of CD103^+^ DCs in generating CD8^+^ T cell responses (175). The contrasting immunogenic and tolerogenic responses are further consistent with the targeting of cDC1s *via* receptors such as DEC-205, where the presence of adjuvant determines the immunogenic or tolerogenic nature of the response (93–95, 97). No markers have yet been identified to distinguish lymphoid resident and migratory cDC2s. Further delineation of resident and migratory DCs and their functions have been reviewed in depth (31, 87, 169); however in the context of targeted vaccines, DCs present at the site of vaccination that are primed to migrate prior to antigen encounter should be considered and further studied.

One unique tissue-resident class of DCs are the Langerhans cells (LCs). LCs are specialized, skin-resident, immature APCs that reside in the skin and can be identified by the presence of cytoplasmic organelles, known as Birbeck granules (176–181). They are also distinguished by the expression of CD207 (Langerin) (176–181). While Langerhans cells share common features of DCs, including the characteristic dendrite projections and expression of CD11c and MHC II, they arise from macrophage progenitors and monocytes (176–178, 180, 181). Thus, while LCs have been classically considered to be a DC subset, some have proposed considering LCs as a macrophage subset (179, 181). Furthermore, studies have observed CD207-expressing DCs that are distinct from LCs, making CD207 a less reliable LC target (177, 178, 180). Regardless, as LCs exist in the skin and encounter vaccine antigens delivered subcutaneously, some studies have investigated targeting vaccines to LCs or CD207^+^ DCs (182). Li et al. targeted LCs using mannose ligands, which bind CD207, conjugated to melanoma antigens. In this study, poor induction of a CD8^+^ T cell response compared to untargeted antigen was observed (183). Bellmann et al. conjugated viral proteins to anti-CD207 or anti-DEC-205 mAbs and directly compared their efficacy in promoting T cell responses in human *ex vivo* skin models (184). Similarly, the CD207-targeted vaccines were ineffective in generating CD8^+^ or CD4^+^ T cell responses, while the DEC-205-targeted formulation generated a moderate T cell response as reported by others (184). These results are consistent with previous findings that dermal DCs, and not LCs, are the primary drivers of immune responses in skin (185). Despite these findings, Carter et al. hypothesize that Th1-biased immune responses are driven by Langerhans cells in the skin (131). They show that CD8^+^ T cell responses are enhanced when targeting cDC2s *via* Dectin-1, which is also expressed by LCs, after subcutaneous injection (131, 146). Given the clear challenges in classifying migratory and resident DCs in the skin, further investigation is required to understand the relative contributions of dermal DCs and Langerhans cells in vaccine-mediated immunity after subcutaneous injection.

### 2.2 Monocytes and monocyte-derived DCs

Monocytes circulate through lymphoid and peripheral tissues, and are recruited to sites of inflammation where they differentiate into monocyte-derived dendritic cells (moDCs) or macrophages (35, 36, 38). Monocytes are typically identified by expression of CD14, CD16 (FcγRIII), CD88, M-CSFR (CD115), F4/80, CD11b, CX3CR1, and Ly6C in mice, and by CD14, CD16, CD88, and CD89 in humans (35, 36, 38, 186–189). Huang et al. demonstrated that monocytes loaded *ex vivo* with tumor antigens and transferred into tumor-bearing mice were able to generate anti-tumor CD8^+^ T cell responses and suppress tumor growth (190). However, Huang et al. also suggested that monocytes themselves have poor antigen presentation capabilities, and that they instead transfer antigen to endogenous APCs for T cell activation (190). *In vivo*, monocytes have been targeted *via* CD11b and Ly6C (191, 192). Lee et al. used a tetrazine-transcyclooctene (TCO) click chemistry strategy to deliver chemotherapeutics to tumor-recruited monocytes. In this study, they first administered TCO-modified anti-CD11b mAbs and then treated with tetrazine-modified mesoporous silica nanoparticles (191). However, CD11b appears on other innate immune cells, and its specificity for monocytes is a limitation of this study. *In vivo* monocyte targeting has also been used for treatment of inflammatory and autoimmune disorders (38). In one example of this approach, Veiga et al. delivered IL-10-encoding mRNA to Ly6C^+^ leukocytes for colitis treatment using lipid nanoparticles (LNPs) bearing a Ly6C scFv targeting moiety (192). However, to date, no *in vivo* targeting of monocytes specifically for vaccine delivery have been reported.

After inflammatory stimulus, monocytes may differentiate into moDCs, and are identified by expression of monocyte markers as well as CD11c, MHC II, CD206, CD24, CD172a (SIRPα), DC-SIGN, and CD64 (FcγRI) (36, 37, 39). Like monocyte targeting, to date, *in vivo* targeting of moDCs for vaccination has not been reported. However, Segura et al. showed that moDCs could generate Th17 responses *ex vivo* (39). As Th17 responses play a critical role in effective vaccination against extracellular bacteria including *M. tuberculosis*, *S. pneumonia*, *B. pertussis*, and *H. pylori* (193), further research in this area is warranted.

### 2.3 Macrophages

In addition to DCs, macrophages are a critical APC involved in adaptive immune responses. Macrophages reside in lymphoid and peripheral tissues, are generally responsible for phagocytosis of pathogens and cellular debris, and can present antigens *via* MHC molecules (194–196). Macrophages are typically identified by the expression of CD11b, F4/80 (mouse)/EMR1 (human), MHC II, CD18 (MAC-1), and CD68 (197–200). It is important to note that, while macrophages can arise from circulating monocytes during inflammation, tissue resident macrophages also arise during embryonic development and are maintained locally (35, 38, 197, 201, 202). Thus, tissue resident and circulating macrophages may not share common monocyte markers. Furthermore, macrophages can exhibit tissue-specific marker expression, similar to DCs (198, 201). We will focus on broad macrophage targeting, as well as specific macrophage phenotypes that can potentially improve vaccine responses, in this review.

Due to their phagocytic activity, macrophages can be passively targeted with micro- or nanoscale carriers. The size, charge, and morphology of micro/nanocarriers affect macrophage uptake. In general, carriers between 100 nm to 3 µm are preferentially taken up by macrophages, highly charged carriers (positive or negative) are preferentially taken up compared to near neutral charges, and spherical carriers are preferentially taken up compared to cylindrical or rod-shaped carriers (203, 204). It should further be noted that the physicochemical properties of these carriers play a key role in downstream responses (205). Active targeting of murine macrophages has been achieved using anti-mouse F4/80 Fab-decorated PLA nanoparticles (206). In one study, Laroui et al. administered these nanoparticles orally to deliver siRNA which inhibited TNF-α production in colonic macrophages for the treatment of colitis (206). While this study specifically sought to invoke a response in the colon, this strategy may be adaptable for pan-macrophage targeting by using alternative routes of administration or modified particle formulations.

#### 2.3.1 M1/M2 macrophage polarization

Macrophages are often classified into “pro-inflammatory” M1 and “anti-inflammatory” M2 phenotypes; M1 macrophages express high levels of MHC II, CD40, CD80, CD86, and TLR4, while M2 macrophages express CD163, CD206, MARCO (CD204), Dectin-1 (Clec7a), and DC-SIGN (CD209) (199, 207, 208). However, these phenotypes are no longer considered discrete states but rather a spectrum, as macrophages may switch from one phenotype to another or simultaneously express markers that define M1 and M2 states (35, 197, 199–201, 203, 204, 207, 208). M1 macrophage targeting has generally focused on polarization towards M2, particularly for applications in treating inflammatory diseases (209). Conversely, M2 targeting has focused on polarization towards M1 or specific M2 depletion. Such targeted responses are of value for cancer therapy, as tumor-associated macrophages (TAMs) are phenotypically similar to M2 macrophages (208, 210), and increased levels of M2 TAMs are associated with poor outcomes (203). However, since “repolarization” strategies often focus on bulk macrophage populations and given the plasticity of the M1/M2 spectrum, specific M1/M2 targeting has, to date, been rare.

We highlight here some examples of macrophage targeting, which have primarily been used for macrophage reprogramming. Jain et al. demonstrated reprogramming of M1 macrophages to M2 using IL-10 encoding DNA loaded into alginate nanoparticles bearing tuftsin peptide, which broadly enhances phagocytosis by macrophages (211, 212). Farajzadeh et al. and Tran et al. demonstrated reprogramming by delivering M2-polarizing therapeutics in hyaluronic acid-based nanoparticles, which bind CD44, a marker overexpressed on macrophages (213, 214). However, both these strategies rely on pan-macrophage marker targeting strategies, rather than targets that achieve M1 or M2-specific delivery.

Alvarado-Vasquez et al. reprogrammed M1 macrophages to M2 by delivering CD163 encoding plasmids *via* a mannose-grafted polyethyleneimine (PEI) nanoparticle, targeting CD206 (215). Conversely, Zhu et al. targeted M2 TAMs using a mannose-grafted PLGA nanoparticle (216), demonstrating the difficulty of M1/M2 target specificity. Similarly, although Dectin-1 is considered a M2 marker, β-1,3-D-glucan nanoparticles targeting Dectin-1 have been used to deliver TNF-α-suppressing siRNAs to pro-inflammatory macrophages (217, 218). However, Cieslewicz et al. demonstrated specific targeting of M2 TAMs, generating a M2-specific targeting peptide by phage display against M2 macrophages to deliver pro-apoptotic peptides (210). This targeting ability and specific depletion could be improved by multivalency (219). Lee et al. also demonstrated M2-specific targeting and depletion using melittin, an amphiphilic peptide derived from honey bee venom, after conjugation to pro-apoptotic peptides (220, 221). M2 TAMs are also known to overexpress folate receptor beta (FRβ), and its natural ligand, folic acid, may be used for M2 TAM targeting (222). Hattori et al. and Tie et al. demonstrated that folate-decorated liposomes were able to target M2 TAMs, while Nagai et al. demonstrated M2 TAM targeting using an FRβ ScFv (223–225). Due to the focus of the field being on altering M1/M2 phenotype switching or M2 depletion, rather than targeting native M1 or M2 macrophages, the effect of M1/M2 targeting on adaptive immune responses remaining an open question and an opportunity for future research.

#### 2.3.2 CD169^+^ macrophages

Another macrophage subset with implications for targeted vaccines is CD169^+^ macrophages. CD169 (Siglec-1) is a sialic acid binding receptor expressed on macrophages from lymphoid tissues (226). CD169^+^ macrophages share the common macrophage markers except for F4/80, which is only expressed on some CD169^+^ macrophages (207). CD169 is expressed on marginal zone macrophages in the spleen, and subcapsular sinus macrophages in the lymph node (207). These macrophages line the barrier between draining blood or lymph and lymphocytes, and can capture draining antigens, suggesting they could play a critical role as vaccine targets. However, CD169^+^ macrophages themselves are poorly phagocytic; rather than presenting antigen, they shuttle intact antigens to B cell follicles to generate antibody responses (227, 228). Alternatively, CD169^+^ macrophages have been shown to transfer antigen to cDC1s to generate CD8^+^ T cell responses, suggesting they are important for all immune responses (229). Because of their selective expression in lymphoid tissues and role in coordinating both B and T cell responses, CD169^+^ macrophages may be a desirable target for targeted vaccines.

While targeting CD169^+^ macrophages is a new concept in vaccine design, a few studies have demonstrated the potential of this approach in mice. van Dinther et al. showed that targeting of OVA to CD169^+^ macrophages using anti-CD169 mAb-antigen conjugates resulted in anti-tumor CD8^+^ T cell responses and slowed progression of OVA-bearing tumors (230). Nijen Twilhaar et al. targeted CD169^+^ macrophages using the liposomes decorated with the ganglioside GM3, a natural ligand for CD169. GM3 liposomes loaded with OVA peptides were able to raise both CD8^+^ and CD4^+^ T cell responses (231). Edgar et al. instead targeted CD169^+^ macrophages with liposomes decorated with a synthetic glycan that binds CD169, termed CD169L (232, 233). CD169L liposomes loaded with OVA alone generated CD4^+^ T cell responses. However, when a mix of TLR7 agonist-loaded and OVA-loaded CD169L liposomes were used, both CD8^+^ and CD4^+^ T cell responses were generated, suggesting that CD169 targeting can be used to modulate CD8^+^ and CD4^+^ T cell ratios (233). In CD169^-/-^ mice, both T cell responses were diminished, indicating the dependence of these responses on CD169. Interestingly, Lisk et al. showed the antibody production of TLR adjuvant-based vaccines was diminished in CD169^-/-^ mice, suggesting the importance of CD169 in TLR-based vaccines (234). Overall, given the importance of CD169^+^ macrophages in shuttling antigen into lymph nodes, targeting them may represent a useful strategy in improving cellular and humoral responses to vaccines.

### 2.4 Granulocytes

Granulocytes are a critical component of the innate immune response against pathogens. These cells, which include neutrophils, eosinophils, basophils, and mast cells, release inflammatory molecules that both directly neutralize pathogens and inform downstream adaptive responses. In addition, granulocytes have been observed to exhibit APC-like characteristics, such as the expression of MHC II and costimulatory markers, under inflammatory conditions. This concept has been extensively reviewed by others (28, 235–238). Granulocytes can inform a wide range of adaptive immune responses. For example, neutrophils can generate Th1 and Th17 biases, while eosinophils and basophils can generate a Th2 bias (28, 239, 240). While the functions and consequences of granulocytes as APCs require further research, they are an understudied aspect of innate immunity with respect to targeted vaccines. Here we will discuss targeting strategies for granulocytes that may potentially be applied to targeted vaccine design.

#### 2.4.1 Neutrophils

Neutrophils comprise most circulating granulocytes. They are recruited to sites of inflammation by chemokines and cytokines, where they release reactive oxygen species (ROS) and immunostimulatory molecules (238). They may also form neutrophil extracellular traps (NETs), web-like structures of DNA that trap and kill pathogens (241, 242). Neutrophils are typically identified by their expression of CD11b and Ly6G (Gr-1) (241, 242). Targeting of neutrophils has been widely studied for the treatment of inflammatory and autoimmune diseases rather than vaccination. Wang et al. and Zhang et al. showed that albumin nanoparticles can passively target activated neutrophils; however, this targeting effect was dependent on nanoparticle opsonization and inflammation-driven upregulation of FcγRII and FcγRIII on neutrophils (243, 244). Active targeting of neutrophils has been accomplished using both antibody and peptide ligands. Vij et al. used PLGA nanoparticles conjugated to an anti-Ly6G/6C antibody to deliver anti-inflammatory drugs to neutrophils (245). A limitation of this targeting strategy is that Ly6C is not specific to neutrophils and could also target other innate immune cells, such as monocytes and monocyte-derived cells. Cruz et al. developed a liposome which used Ly6G Fabs for neutrophil-specific targeting in order to deliver drugs that modulate neutrophil activity (246). Further specificity was conferred by adding a peptide derived from α1-antitrypsin, the natural ligand for neutrophil elastase, a surface protein exclusively expressed on activated neutrophils (246, 247). Volz et al. generated a neutrophil-specific targeting peptide by phage display which binds CD177, a neutrophil-specific activation marker. This CD177-targeting peptide was used to deliver PLGA nanoparticles to neutrophils containing immunomodulatory molecules (248).

For targeted vaccines, almost no studies have directly measured the response of targeting vaccines to neutrophils. It is known that alum based vaccines recruits neutrophils to injection sites and other large positively charged nanocarriers also induce neutrophil recruitment, which is especially helpful for generation of antibacterial Th1/Th17 responses (239, 240, 249, 250). It may be possible that neutrophils assist in this Th1/Th17 response, but further research is required to determine how targeting neutrophils effects vaccination efficacy.

#### 2.4.2 Eosinophils, basophils, and mast cells

Eosinophils, basophils, and mast cells comprise the remaining populations of granulocytes. They are generally involved in Th2 responses, and release immunostimulatory molecules upon activation. Eosinophils are activated by IL-5 in response to allergy or parasitic infections, while basophils and mast cells degranulate in response to crosslinking of surface-bound IgE and activation of the high-affinity Fc receptor for IgE (FcϵR1) (238). Eosinophils are typically identified by expression of CD11b, CCR3, and Siglec-F (mouse)/Siglec-8 (human) (251). Basophils and mast cells share the markers CD11b, IL3Ra (CD123), FcϵR1, and CD203c (ENPP-3), with basophils additionally expressing CD49b and mast cells additionally expressing c-kit (CD117) (252, 253).

Eosinophils, basophils, and mast cells have generally been targeted for the treatment of allergic diseases. Siglec-F/Siglec-8 is a common eosinophil target, and Siglec-8 antibody targeting for eosinophil depletion has been used in clinical trials to treat eosinophilic disorders (254). For targeted delivery to eosinophils, Nycholat et al. developed synthetic glycans that bind both Siglec-F/Siglec-8, and showed that liposomes decorated with these glycans effectively trafficked to eosinophils *in vivo* (255). In humans, basophils and mast cells express Siglec-3 (CD33) (256), and Duan et al. showed that liposomes decorated with a synthetic glycan targeting Siglec-3 were able to target human mast cells in a CD33-transgenic mouse model (257, 258). Targeting of basophils and mast cells can also be achieved through the exclusively expressed membrane-associated protein CD203c. Gold nanoparticles functionalized with anti-CD203c antibodies were shown to bind human basophil and mast cells *in vitro*; however, *in vivo* targeting has yet to be demonstrated with this system (259, 260). As with neutrophils, these granulocytes are also understudied for their participation in vaccination responses, particularly vaccines engineered for type 2 immune responses.

### 2.5 Lymph node targeting and non-innate immune cell targeting

A crucial factor in vaccine design is lymphatic targeting. Lymph is composed of draining interstitial fluid, carrying antigens and immune cells to the lymph nodes as a constant form of immune surveillance. Lymph nodes are therefore critical sites for coordination of both antibody and T cell responses, and targeting immune cell subsets that reside in, or traffic to, lymph nodes are potent methods to improve vaccine-induced immunity.

Typical strategies for broad lymphatic delivery include nanoparticles and albumin “hitchhiking” (261–265). Size is one of the primary determinants of lymphatic entry, and nanomaterials on the scale of 100 nm preferentially drain from the interstitium and into lymphatics (264, 266). Albumin is an abundant protein that constantly circulates through blood, exits into the interstitium, then drains into the lymph *via* active transcellular or passive paracellular mechanisms before returning to blood circulation (263). Thus, albumin-binding or albumin-complexed therapeutics may also access lymphatics. Strategies for broad lymphatic targeting have been extensively reviewed (261–265); this review will focus on active targeting of specific lymph-node associated cells for vaccine-induced immunity.

Many of the innate immune cell subsets described in this review are present in lymph nodes, and active targeting may be paired with lymphatic delivery to achieve lymph node cell-specific targeting. However, several other types of cells coordinate with these innate immune cells to generate vaccine-induced immune responses. For example, while the main function of B cells is their adaptive immune response, particularly their differentiation into antibody-producing cells and the generation of humoral responses, B cells are also professional APCs capable of presenting antigens as peptide-MHC complexes (44). Additionally, lymph node stromal cells (LNSCs) provide the architecture and support required for innate and adaptive immune cell responses, and they can also play critical immunomodulatory roles themselves. While the subsets and functions of LNSCs have been extensively reviewed elsewhere (246, 247), here we will focus on two LNSC subsets: follicular dendritic cells (FDCs) and lymphatic endothelial cells (LECs). While B cells, FDCs, and LECs do not belong to the innate immune cell compartment, this section will discuss both the ability of these cells to improve adaptive immune responses for vaccination and methods of targeting these cells.

### 2.6 B cells

While one of the main functions of B cells is their adaptive immune response, mainly their differentiation into antibody-producing cells and the generation of humoral responses, B cells are also professional APCs, capable of presenting antigens as peptide-MHC II complexes (44). B cells are typically identified by their expression of CD19, CD20, B220, MHC II, and CD21/35 (CR1/2) (44). As B cells reside in lymphoid tissues, they also provide a measure of lymph node targeting.

B cells are organized into follicles within lymphoid tissues and may be passively targeted with vaccine nanoformulations. Nanoformulations on the scale of 100 nm preferentially drain to lymph nodes, where they may directly access B cell follicles or be transferred into follicles by CD169^+^ subcapsular sinus macrophages in a CD21/35-dependent manner (227, 228, 261, 267). Shen et al. demonstrated that iron nanoparticles were rapidly opsonized by complement component 3b (C3b), allowing nanoparticles to be taken up by B cells *via* CD21/35 (268). Thus, while nanocarrier targeting of B cells is considered “passive” targeting, from another viewpoint, intentional complement opsonization of nanocarriers and uptake *via* CD21/35 could be considered an “active” targeting approach. Using this principle, Shimizu et al. used polyethylene glycol (PEG)-decorated (PEGylated) liposomes to induce a weak anti-PEG IgM response (269–271). Upon administration of a second, PEGylated liposome loaded with antigen, liposomes were opsonized with IgM and complement proteins, allowing B cell uptake and adaptive immune responses toward the antigen (269–271). Further chemical modification of PEG to contain terminal hydroxyl groups, which engage the complement system, allowed for B cell targeting with PEGylated liposomes in a single injection (272).

Since B cells initiate humoral responses after binding antigens with their B cell receptors (BCRs), antigens themselves may be considered a form of antigen-specific B cell targeting. Multivalent antigen display on nanocarriers has also been shown to enhance BCR crosslinking, improving humoral responses (261). Temchura et al. demonstrated that calcium phosphate (CaP) nanoparticles coated with antigens and adjuvants were able to target and activate B cells *in vitro* (273). *In vivo* B cell targeting led to CD4^+^ T cell and humoral responses (274). Moyer et al. demonstrated that antigens adsorbed to aluminum hydroxide (alum), which acts as both a nanocarrier and adjuvant, was taken up by and strongly activated B cells *in vivo* (275). The alum-adsorbed antigen formulation enhanced antibody levels relative to non-adsorbed antigens. It is important to consider that in both these cases, the targeted B cells were transgenic, antigen-specific cells. This makes it difficult to assess if BCR targeting is generalizable to other antigens and endogenous antigen-specific B cells. Despite this, taken together these studies indicate that nanocarrier targeting and multivalent antigen display are effective strategies for B cell-targeted vaccines.

B cells may also be actively targeted *via* their surface receptors CD19 and MHC II. CD19 is a membrane protein that reduces the threshold for BCR activation (276, 277). Yan et al. compared the targeting of B cells *via* anti-CD19 and anti-IgM mAb-antigen conjugates. Both CD19 targeting and IgM targeting were able to deliver antigens to B cells and induced T cell activation *in vivo* (278). Ma et al. and Ding et al. further showed that targeting tumor antigens to B cells *via* CD19 induced strong antibody responses, and both CD8^+^ and CD4^+^ cell responses, which were able to suppress tumor growth (279, 280). Instead of targeting CD19, Andersen et al. and Hinke et al. targeted B cells *via* MHC II using DNA vaccines that expressed multivalent HA fused to anti-MHC II scFvs. MHC II targeting improved antibody and Tfh responses compared to non-targeting formulations, while multivalency improved for protection against flu challenge compared to single antigen formulations, likely through increased BCR crosslinking (281, 282). Although MHC II is also expressed on other APCs, limiting B cell-specific targeting, Andersen et al. showed that DC presentation was not required for the magnitude of antibodies produced by B cell targeting in their vaccine (281). Taken together, targeting B cells in a vaccination is a promising approach for the induction of strong antibody responses. Further study is required to understand the relative contributions of B cell targeting to T cell responses, such as CD4^+^ Tfh and type 2 immune responses, and cellular-biased T cell responses, such as CD8^+^ T cell responses, in vaccination.

### 2.7 Lymph node stromal cells

Lymph node stromal cells (LNSCs) provide the architecture and support required for innate and adaptive immune cell responses, but also play critical immunomodulatory roles themselves. While the subsets and functions of LNSCs have been extensively reviewed elsewhere (246, 247), here we will focus on two LNSC subsets, follicular dendritic cells (FDCs) and lymphatic endothelial cells (LECs).

#### 2.7.1 Follicular dendritic cells

Despite sharing a name with DCs, follicular dendritic cells (FDCs) are a functionally distinct cell subset with a key role in adaptive immunity. FDCs arise from mesenchymal, rather than hematopoietic progenitors, only sharing the characteristic dendrite morphology (283, 284). FDCs are a rare population that reside in the B cell follicles, but do not process antigen. Instead, FDCs retain antigens in native form *via* immune complexes for presentation to germinal center B cells (283–286). B cells with high-affinity, antigen-specific BCRs bind retained antigen, and receive survival signals from FDCs, a critical process known as affinity maturation (283–286). Thus, vaccines targeting FDCs may be able to generate robust humoral responses.

FDCs are typically identified by expression of gp38 (PDPN) and CD21/35 (285, 286). Given their location in B cell follicles, targeting strategies that utilize opsonization and CD21/35 binding for B cell targeting may also be applicable to FDCs. Zhang et al. showed that both 15-50 nm-sized and 50-100 nm-sized antigen-conjugated gold nanoparticles were able to leverage lymphatic transport and traffic to lymph nodes (287). However, 50-100 nm-sized nanoparticles preferentially accumulated on FDCs due to increased C3b opsonization and CD21/35 binding (287). Mattsson et al. and Schussek et al. showed that a CD21/35-binding adjuvant derived from cholera toxin bound to FDCs, and strongly potentiated antibody and Tfh responses (288, 289). Aung et al. demonstrated that FDCs could be targeted with antigens fused to anti-CD35 ScFvs (290). After vaccination, targeted antigen was retained on FDCs longer, and significantly increased antigen-specific IgG, compared to a non-targeted control (290). Given the importance of FDCs to mature antibody production and humoral immune responses, further research on antigen targeting to FDCs is warranted.

#### 2.7.2 Lymphatic endothelial cells

LECs line the lymphatic vessels and lymph nodes. As a result, they are poised to interact with antigens and immune cells as they enter lymphatic circulation. LECs are capable of antigen capture and presentation to T cells (291–298). Under steady-state conditions, LECs are also known to generate long-lived memory CD8^+^ T cells that can differentiate into effector cells after re-challenge (294). Under inflammatory conditions such as viral infections, antigen persistence in LECs has been observed in a mechanism distinct from FDC retention (291–293). This persistence enhanced memory CD8^+^ T cell responses against infections. Thus, vaccines targeting LECs may be able to generate robust memory T cell responses.

LECs are typically identified by expression of gp38 (PDPN), CD31, LYVE1, and VEGFR3 (299, 300). While no LEC-targeting vaccine formulations have been reported, Guo et al. demonstrated that LEC-targeting magnetic nanoparticles for magnetic resonance imaging (MRI), functionalized with anti-LYVE-1 mAbs, were able to target LECs *in vitro* (301). Wu et al. further showed that magnetic nanoparticles functionalized with both anti-LYVE-1 and anti-gp38 mAbs were able to target LECs *in vivo* (302). While targeting systems exist, further research will also be required to show how selectively targeting vaccines to LECs affects adaptive responses.

## 3 Discussion

This review presents the numerous strategies for vaccine targeting of specific cell subsets and the implications on adaptive immune responses. Through compiling these studies, several trends could be observed. For example, general targeting of cDC1 regardless of receptor generated stronger CD8^+^ T cell responses compared to other cell types. However, cDC1s did not exclusively generate CD8^+^ T cell responses, as targeting of receptors such as Clec9a could result in humoral and Tfh responses. Neither were CD8^+^ T cell responses exclusive to cDC1s, as cDC2s could additionally generate CD8^+^ T cell responses in addition to CD4^+^ T cell responses. The type of CD4^+^ T cell response also varied widely depending on the target receptor, although the relatively fewer number of cDC2-specific receptors should be considered in interpreting these results. Immune cells such as monocytes, macrophages, B cells, neutrophils, and other granulocytes have been targeted in a wide variety of therapeutics, but their roles as target cells in vaccine delivery are still poorly defined. However, the fundamental hypothesis that the cells that first encounter vaccine material impacts adaptive immunity, is confirmed by a large body of literature. This review also presents stromal cells, such as FDCs and LECs, as new opportunities for targeted vaccine research that could potentially generate long-lived humoral and cellular responses.

It is important to note that there are other design considerations for cell targeted vaccines not discussed in this review, including immune responses against targeting constructs and anti-carrier immunity, off-target effects, and cell receptor disruption. For example, in murine studies, the targeting antibodies utilized are often of rat or hamster origin, which can lead to an anti-antibody host response due to non-self immune recognition (303–305). Repeated administration, which may be required in some vaccine schedules, can result in either immunogenic or tolerogenic responses toward these antibodies, but also toward any carrier molecules (306). Anti-host antibodies have been used as a readout for immunological outcomes of targeted vaccines after injection of the targeting antibody alone. However, when antibodies or other biologics are used to target antigens to specific cells, it is necessary to decouple the immune reactions to the antigen versus the targeting component. This could be accomplished by analyzing antigen-specific cellular and humoral responses. In human and clinical studies, these issues are often pre-empted by using chimeric, humanized, or fully human antibodies that reduce the risk of anti-antibody responses (303–306). However, the potential for non-antigen specific immune responses should still be considered. Another important consideration is the potential for cell signaling after engagement of a target molecule. For example, some target receptors (e.g. FcγRs, DCIR) contain ITAMs or ITIMs, or are costimulatory molecules (e.g. CD40), which may induce or restrict downstream immune responses. In the case of FcγR, no differences in immune responses were observed between targeting of activating or inhibitory isoforms (105). However, to fully understand the immunological effects of targeted vaccines, it is critical to evaluate if the targeting method can induce any agonistic or antagonistic effects after ligation of the target receptor, which may alter an immune response. Some targets are ITAM and ITIM-independent (e.g. DEC-205), or may have pleiotropic effects depending on if they are targeted under inflammatory or steady state conditions (88, 94). Lastly, while some receptors are highly specific to a cell subtype, many receptors mentioned in this review are present on many innate immune cell types, and targeting these receptors can lead to off-target cellular activation and impact vaccine efficacy.

Here we have shown how control over innate immune response can be achieved by targeting vaccines to specific cellular subsets or surface markers. This body of literature will be useful for the design of next generation vaccines which carefully match desired adaptive immune response to specific diseases.

## Author contributions

The idea for this review was conceived by PD. PD generated the figure. PD, TU, AMW and NSR contributed to reviewing literature, formatting the article, writing of the text and critique of the text. APE-K provided intellectual oversight, review, and critique of the article. All authors contributed to the article and approved the submitted version.

## References

[1] Moyle P Toth I. Self-adjuvanting lipopeptide vaccines. Curr Med Chem (2008) 15:506–16. doi: 10.2174/092986708783503249 18289006

[2] Moyle PM Toth I. Modern subunit vaccines: development, components, and research opportunities. ChemMedChem (2013) 8:360–76. doi: 10.1002/cmdc.201200487 23316023

[3] Pollard AJ Bijker EM. A guide to vaccinology: from basic principles to new developments. Nat Rev Immunol (2021) 21:83–100. doi: 10.1038/s41577-020-00479-7 33353987 PMC7754704

[4] Kobiyama K Ishii KJ. Making innate sense of mRNA vaccine adjuvanticity. Nat Immunol (2022) 23:474–6. doi: 10.1038/s41590-022-01168-4 35354958

[5] Shan C Xie X Shi P-Y. Zika virus vaccine: progress and challenges. Cell Host Microbe (2018) 24:12–7. doi: 10.1016/j.chom.2018.05.021 PMC6112613 30008291

[6] Chaudhary N Weissman D Whitehead KA. mRNA vaccines for infectious diseases: principles, delivery and clinical translation. Nat Rev Drug Discovery (2021) 20:817–38. doi: 10.1038/s41573-021-00283-5 PMC8386155 34433919

[7] Janeway CA Medzhitov R. Innate immune recognition. Annu Rev Immunol (2002) 20:197–216. doi: 10.1146/annurev.immunol.20.083001.084359 11861602

[8] Takeuchi O Akira S. Pattern recognition receptors and inflammation. Cell (2010) 140:805–20. doi: 10.1016/j.cell.2010.01.022 20303872

[9] Pashine A Valiante NM Ulmer JB. Targeting the innate immune response with improved vaccine adjuvants. Nat Med (2005) 11:S63–8. doi: 10.1038/nm1210 15812492

[10] Akira S Hemmi H. Recognition of pathogen-associated molecular patterns by TLR family. Immunol Lett (2003) 85:85–95. doi: 10.1016/S0165-2478(02)00228-6 12527213

[11] Bergmann-Leitner E Leitner W. Adjuvants in the driver’s seat: how magnitude, type, fine specificity and longevity of immune responses are driven by distinct classes of immune potentiators. Vaccines (2014) 2:252–96. doi: 10.3390/vaccines2020252 PMC4494256 26344620

[12] Pulendran B Arunachalam PS O’Hagan DT. Emerging concepts in the science of vaccine adjuvants. Nat Rev Drug Discovery (2021) 20:454–75. doi: 10.1038/s41573-021-00163-y PMC8023785 33824489

[13] Coffman RL Sher A Seder RA. Vaccine adjuvants: putting innate immunity to work. Immunity (2010) 33:492–503. doi: 10.1016/j.immuni.2010.10.002 21029960 PMC3420356

[14] Schijns V Fernández-Tejada A Barjaktarović Ž. Bouzalas I Brimnes J Chernysh S. Modulation of immune responses using adjuvants to facilitate therapeutic vaccination. Immunol Rev (2020) 296:169–90. doi: 10.1111/imr.12889 PMC7497245 32594569

[15] Marshall JS Warrington R Watson W Kim HL. An introduction to immunology and immunopathology. Allergy Asthma Clin Immunol (2018) 14:49. doi: 10.1186/s13223-018-0278-1 30263032 PMC6156898

[16] Krishnaswamy JK Alsén S Yrlid U Eisenbarth SC Williams A. Determination of T follicular helper cell fate by dendritic cells. Front Immunol (2018) 9:2169. doi: 10.3389/fimmu.2018.02169 30319629 PMC6170619

[17] Gong F Zheng T Zhou P. T follicular helper cell subsets and the associated cytokine IL-21 in the pathogenesis and therapy of asthma. Front Immunol (2019) 10:2918. doi: 10.3389/fimmu.2019.02918 31921177 PMC6923700

[18] Zhu X Zhu J. CD4 T helper cell subsets and related human immunological disorders. Int J Mol Sci (2020) 21:8011. doi: 10.3390/ijms21218011 33126494 PMC7663252

[19] Annunziato F Romagnani C Romagnani S. The 3 major types of innate and adaptive cell-mediated effector immunity. J Allergy Clin Immunol (2015) 135:626–35. doi: 10.1016/j.jaci.2014.11.001 25528359

[20] Grover P Goel PN Greene MI. Regulatory T cells: regulation of identity and function. Front Immunol (2021) 12:750542. doi: 10.3389/fimmu.2021.750542 34675933 PMC8524049

[21] Daniel C Wennhold K Kim H-J Von Boehmer H. Enhancement of antigen-specific Treg vaccination in *vivo* . Proc Natl Acad Sci USA (2010) 107:16246–51. doi: 10.1073/pnas.1007422107 PMC2941325 20805478

[22] Thompson MR Kaminski JJ Kurt-Jones EA Fitzgerald KA. Pattern recognition receptors and the innate immune response to viral infection. Viruses (2011) 3:920–40. doi: 10.3390/v3060920 PMC3186011 21994762

[23] Sarkar I Garg R van Drunen Littel-van den Hurk S. Selection of adjuvants for vaccines targeting specific pathogens. Expert Rev Vaccines (2019) 18:505–21. doi: 10.1080/14760584.2019.1604231 PMC7103699 31009255

[24] Tesfaye DY Gudjonsson A Bogen B Fossum E. Targeting conventional dendritic cells to fine-tune antibody responses. Front Immunol (2019) 10:1529. doi: 10.3389/fimmu.2019.01529 31333661 PMC6620736

[25] Cohn L Delamarre L. Dendritic cell-targeted vaccines. Front Immunol (2014) 5:255. doi: 10.3389/fimmu.2014.00255 24910635 PMC4039009

[26] Paul S Lal G. The molecular mechanism of natural killer cells function and its importance in cancer immunotherapy. Front Immunol (2017) 8:1124. doi: 10.3389/fimmu.2017.01124 28955340 PMC5601256

[27] Caron J Ridgley LA Bodman-Smith M. How to train your dragon: harnessing gamma delta T cells antiviral functions and trained immunity in a pandemic era. Front Immunol (2021) 12:666983. doi: 10.3389/fimmu.2021.666983 33854516 PMC8039298

[28] Lin A Loré K. Granulocytes: new members of the antigen-presenting cell family. Front Immunol (2017) 8:1781. doi: 10.3389/fimmu.2017.01781 29321780 PMC5732227

[29] Schuijs MJ Hammad H Lambrecht BN. Professional and ‘Amateur’ Antigen-presenting cells in type 2 immunity. Trends Immunol (2019) 40:22–34. doi: 10.1016/j.it.2018.11.001 30502024 PMC7610811

[30] Banchereau J Briere F Caux C Davoust J Lebecque S Liu Y-J. Immunobiology of dendritic cells. Annu Rev Immunol (2000) 18:767–811. doi: 10.1146/annurev.immunol.18.1.767 10837075

[31] Merad M Sathe P Helft J Miller J Mortha A. The dendritic cell lineage: ontogeny and function of dendritic cells and their subsets in the steady state and the inflamed setting. Annu Rev Immunol (2013) 31:563–604. doi: 10.1146/annurev-immunol-020711-074950 23516985 PMC3853342

[32] Castell-Rodríguez A Piñón-Zárate G Herrera-Enríquez M Jarquín-Yáñez K Medina-Solares I. Dendritic cells: location, function, and clinical implications. In: Ghosh A, editor. Biology of Myelomonocytic Cells. (London, UK: InTech) (2017). Available at: http://www.intechopen.com/books/biology-of-myelomonocytic-cells/dendritic-cells-location-function-and-clinical-implications.

[33] Macri C Pang ES Patton T O’Keeffe M. Dendritic cell subsets. Semin Cell Dev Biol (2018) 84:11–21. doi: 10.1016/j.semcdb.2017.12.009 29246859

[34] Rodrigues PF Tussiwand R. Novel concepts in plasmacytoid dendritic cell (pDC) development and differentiation. Mol Immunol (2020) 126:25–30. doi: 10.1016/j.molimm.2020.07.006 32739721

[35] Epelman S Lavine KJ Randolph GJ. Origin and functions of tissue macrophages. Immunity (2014) 41:21–35. doi: 10.1016/j.immuni.2014.06.013 25035951 PMC4470379

[36] Qu C Brinck-Jensen N-S Zang M Chen K. Monocyte-derived dendritic cells: targets as potent antigen-presenting cells for the design of vaccines against infectious diseases. Int J Infect Diseases (2014) 19:1–5. doi: 10.1016/j.ijid.2013.09.023 24216295

[37] Langlet C Tamoutounour S Henri S Luche H Ardouin L Grégoire C. CD64 expression distinguishes monocyte-derived and conventional dendritic cells and reveals their distinct role during intramuscular immunization. J Immunol (2012) 188:1751–60. doi: 10.4049/jimmunol.1102744 22262658

[38] Chow KV Sutherland RM Zhan Y Lew AM. Heterogeneity, functional specialization and differentiation of monocyte-derived dendritic cells. Immunol Cell Biol (2017) 95:244–51. doi: 10.1038/icb.2016.104 27748730

[39] Segura E Touzot M Bohineust A Cappuccio A Chiocchia G HosMalin A. Human inflammatory dendritic cells induce Th17 cell differentiation. Immunity (2013) 38:336–48. doi: 10.1016/j.immuni.2012.10.018 23352235

[40] Musumeci A Lutz K Winheim E Krug AB. What makes a pDC: recent advances in understanding plasmacytoid DC development and heterogeneity. Front Immunol (2019) 10:1222. doi: 10.3389/fimmu.2019.01222 31191558 PMC6548821

[41] Collin M Bigley V. Human dendritic cell subsets: an update. Immunology (2018) 154:3–20. doi: 10.1111/imm.12888 29313948 PMC5904714

[42] Swiecki M Colonna M. The multifaceted biology of plasmacytoid dendritic cells. Nat Rev Immunol (2015) 15:471–85. doi: 10.1038/nri3865 PMC4808588 26160613

[43] Sichien D Lambrecht BN Guilliams M Scott CL. Development of conventional dendritic cells: from common bone marrow progenitors to multiple subsets in peripheral tissues. Mucosal Immunol (2017) 10:831–44. doi: 10.1038/mi.2017.8 28198365

[44] Adler LN Jiang W Bhamidipati K Millican M Macaubas C Hung S. The other function: class II-restricted antigen presentation by B cells. Front Immunol (2017) 8:319. doi: 10.3389/fimmu.2017.00319 28386257 PMC5362600

[45] Wang Q Cao W Yang Z-G Zhao G-F. DC targeting DNA vaccines induce protective and therapeutic antitumor immunity in mice. Int J Clin Exp Med (2015) 8:17565–77. PMC4694247 26770347

[46] van Broekhoven CL Parish CR Demangel C Britton WJ Altin JG. Targeting dendritic cells with antigen-containing liposomes. Cancer Res (2004) 64:4357–65. doi: 10.1158/0008-5472.CAN-04-0138 15205352

[47] Lewis JS Zaveri TD Crooks CP Keselowsky BG. Microparticle surface modifications targeting dendritic cells for non-activating applications. Biomaterials (2012) 33:7221–32. doi: 10.1016/j.biomaterials.2012.06.049 PMC3428206 22796161

[48] Yi S Zhang X Sangji MH Liu Y Allen SD Xiao B. Surface engineered polymersomes for enhanced modulation of dendritic cells during cardiovascular immunotherapy. Adv Funct Mater (2019) 29:1904399. doi: 10.1002/adfm.201904399 34335131 PMC8320590

[49] Liu Y Yao L Cao W Liu Y Zhai W Wu Y. Dendritic cell targeting peptide-based nanovaccines for enhanced cancer immunotherapy. ACS Appl Bio Mater (2019) 2:1241–54. doi: 10.1021/acsabm.8b00811 35021373

[50] Puth S Verma V Hong SH Tan W Lee SE Rhee JH. An all-in-one adjuvanted therapeutic cancer vaccine targeting dendritic cell cytosol induces long-lived tumor suppression through NLRC4 inflammasome activation. Biomaterials (2022) 286:121542. doi: 10.1016/j.biomaterials.2022.121542 35594837

[51] Cruz LJ Rosalia RA Kleinovink JW Rueda F Löwik CWGM Ossendorp F. Targeting nanoparticles to CD40, DEC-205 or CD11c molecules on dendritic cells for efficient CD8^+^ T cell response: A comparative study. J Control Release (2014) 192:209–18. doi: 10.1016/j.jconrel.2014.07.040 25068703

[52] Castro FVV Tutt AL White AL Teeling JL James S French RR. CD11c provides an effective immunotarget for the generation of both CD4 and CD8 T cell responses. Eur J Immunol (2008) 38:2263–73. doi: 10.1002/eji.200838302 18651710

[53] Wang H Griffiths MN Burton DR Ghazal P. Rapid antibody responses by low-dose, single-step, dendritic cell-targeted immunization. Proc Natl Acad Sci USA (2000) 97:847–52. doi: 10.1073/pnas.97.2.847 PMC15419 10639168

[54] Dunkelberger JR Song W-C. Complement and its role in innate and adaptive immune responses. Cell Res (2010) 20:34–50. doi: 10.1038/cr.2009.139 20010915

[55] Pugholm LH Petersen LR Søndergaard EKL Varming K Agger R. Enhanced humoral responses induced by targeting of antigen to murine dendritic cells. Scand J Immunol (2015) 82:515–22. doi: 10.1111/sji.12387 26346906

[56] Braathen R Spång HCL Lindeberg MM Fossum E Grødeland G Fredriksen AB. The magnitude and IgG subclass of antibodies elicited by targeted DNA vaccines are influenced by specificity for APC surface molecules. Immunohorizons (2018) 2:38–53. doi: 10.4049/immunohorizons.1700038 31022690

[57] White AL Tutt AL James S Wilkinson KA Castro FVV Dixon SV. Ligation of CD11c during vaccination promotes germinal centreinduction and robust humoral responses without adjuvant. Immunology (2010) 131:141–51. doi: 10.1111/j.1365-2567.2010.03285.x 20465572 PMC2966766

[58] Mountford AP Fisher A Wilson RA. The profile of IgG1 and IgG2a antibody responses in mice exposed to Schistosoma mansoni. Parasite Immunol (1994) 16:521–7. doi: 10.1111/j.1365-3024.1994.tb00306.x 7870462

[59] Zani I Stephen S Mughal N Russell D Homer-Vanniasinkam S Wheatcroft S. Scavenger receptor structure and function in health and disease. Cells (2015) 4:178–201. doi: 10.3390/cells4020178 26010753 PMC4493455

[60] Sallusto F Cella M Danieli C Lanzavecchia A. Dendritic cells use macropinocytosis and the mannose receptor to concentrate macromolecules in the major histocompatibility complex class II compartment: downregulation by cytokines and bacterial products. J Exp Med (1995) 182:389–400. doi: 10.1084/jem.182.2.389 7629501 PMC2192110

[61] Burgdorf S Lukacs-Kornek V Kurts C. The mannose receptor mediates uptake of soluble but not of cell-associated antigen for cross-presentation. J Immunol (2006) 176:6770–6. doi: 10.4049/jimmunol.176.11.6770 16709836

[62] Chatterjee B Smed-Sörensen A Cohn L Chalouni C Vandlen R Lee B-C. Internalization and endosomal degradation of receptor-bound antigens regulate the efficiency of cross presentation by human dendritic cells. Blood (2012) 120:2011–20. doi: 10.1182/blood-2012-01-402370 22791285

[63] He L-Z Weidlick J Sisson C Marsh HC Keler T. Toll-like receptor agonists shape the immune responses to a mannose receptor-targeted cancer vaccine. Cell Mol Immunol (2015) 12:719–28. doi: 10.1038/cmi.2014.100 PMC4716615 25345808

[64] Wilson DS Hirosue S Raczy MM Bonilla-Ramirez L Jeanbart L Wang R. Antigens reversibly conjugated to a polymeric glyco-adjuvant induce protective humoral and cellular immunity. Nat Mater (2019) 18:175–85. doi: 10.1038/s41563-018-0256-5 30643235

[65] Singh SK Streng-Ouwehand I Litjens M Kalay H Burgdorf S Saeland E. Design of neo-glycoconjugates that target the mannose receptor and enhance TLR-independent cross-presentation and Th1 polarization. Eur J Immunol (2011) 41:916–25. doi: 10.1002/eji.201040762 21400496

[66] He L-Z Crocker A Lee J Mendoza-Ramirez J Wang X-T Vitale LA. Antigenic targeting of the human mannose receptor induces tumor immunity. J Immunol (2007) 178:6259–67. doi: 10.4049/jimmunol.178.10.6259 17475854

[67] MacDonald KPA Munster DJ Clark GJ Dzionek A Schmitz J Hart DNJ. Characterization of human blood dendritic cell subsets. Blood (2002) 100:4512–20. doi: 10.1182/blood-2001-11-0097 12393628

[68] Mnich ME van Dalen R van Sorge NM. C-type lectin receptors in host defense against bacterial pathogens. Front Cell Infect Microbiol (2020) 10:309. doi: 10.3389/fcimb.2020.00309 32733813 PMC7358460

[69] Segura E Valladeau-Guilemond J Donnadieu M-H Sastre-Garau X Soumelis V Amigorena S. Characterization of resident and migratory dendritic cells in human lymph nodes. J Exp Med (2012) 209:653–60. doi: 10.1084/jem.20111457 PMC3328358 22430490

[70] Askew D Harding CV. Antigen processing and CD24 expression determine antigen presentation by splenic CD4^+^ and CD8^+^ dendritic cells. Immunology (2008) 123:447–55. doi: 10.1111/j.1365-2567.2007.02711.x PMC2433328 17949418

[71] Hongo D Zheng P Dutt S Pawar RD Meyer E Engleman EG. Identification of two subsets of murine DC1 dendritic cells that differ by surface phenotype, gene expression, and function. Front Immunol (2021) 12:746469. doi: 10.3389/fimmu.2021.746469 34777358 PMC8589020

[72] Fang X Zheng P Tang J Liu Y. CD24: from A to Z. Cell Mol Immunol (2010) 7:100–3. doi: 10.1038/cmi.2009.119 PMC4001892 20154703

[73] Guilliams M Dutertre C-A Scott CL McGovern N Sichien D Chakarov S. Unsupervised high-dimensional analysis aligns dendritic cells across tissues and species. Immunity (2016) 45:669–84. doi: 10.1016/j.immuni.2016.08.015 PMC5040826 27637149

[74] Steinbrecher A Reinhold D Quigley L Gado A Tresser N Izikson L. Targeting dipeptidyl peptidase IV (CD26) suppresses autoimmune encephalomyelitis and up-regulates TGF-β1 secretion *in vivo* . J Immunol (2001) 166:2041–8. doi: 10.4049/jimmunol.166.3.2041 11160254

[75] Ohnuma K Haagmans BL Hatano R Raj VS Mou H Iwata S. Inhibition of middle east respiratory syndrome coronavirus infection by anti-CD26 monoclonal antibody. J Virol (2013) 87:13892–9. doi: 10.1128/JVI.02448-13 PMC3838260 24067970

[76] Salnikov AV Bretz NP Perne C Hazin J Keller S Fogel M. Antibody targeting of CD24 efficiently retards growth and influences cytokine milieu in experimental carcinomas. Br J Cancer (2013) 108:1449–59. doi: 10.1038/bjc.2013.102 PMC3629417 23511563

[77] Han Y Sun F Zhang X Wang T Jiang J Cai J. CD24 targeting bi-specific antibody that simultaneously stimulates NKG2D enhances the efficacy of cancer immunotherapy. J Cancer Res Clin Oncol (2019) 145:1179–90. doi: 10.1007/s00432-019-02865-8 PMC11810302 30778749

[78] Bosteels C Neyt K Vanheerswynghels M van Helden MJ Sichien D Debeuf N. Inflammatory Type 2 cDCs Acquire Features of cDC1s and Macrophages to Orchestrate Immunity to Respiratory Virus Infection. Immunity (2020) 52:1039–1056.e9. doi: 10.1016/j.immuni.2020.04.005 32392463 PMC7207120

[79] Villani A-C Satija R Reynolds G Sarkizova S Shekhar K Fletcher J. Single-cell RNA-seq reveals new types of human blood dendritic cells, monocytes, and progenitors. Science (2017) 356:eaah4573. doi: 10.1126/science.aah4573 28428369 PMC5775029

[80] Ginhoux F Guilliams M Merad M. Expanding dendritic cell nomenclature in the single-cell era. Nat Rev Immunol (2022) 22:67–8. doi: 10.1038/s41577-022-00675-7 35027741

[81] Durai V Murphy KM. Functions of murine dendritic cells. Immunity (2016) 45:719–36. doi: 10.1016/j.immuni.2016.10.010 PMC5145312 27760337

[82] Anderson DA Dutertre C-A Ginhoux F Murphy KM. Genetic models of human and mouse dendritic cell development and function. Nat Rev Immunol (2021) 21:101–15. doi: 10.1038/s41577-020-00413-x PMC10955724 32908299

[83] Shin J-Y Wang C-Y Lin C-C Chu C-L. A recently described type 2 conventional dendritic cell (cDC2) subset mediates inflammation. Cell Mol Immunol (2020) 17:1215–7. doi: 10.1038/s41423-020-0511-y PMC7785008 32732988

[84] Krishnaswamy JK Gowthaman U Zhang B Mattsson J Szeponik L Liu D. Migratory CD11b^+^ conventional dendritic cells induce T follicular helper cell–dependent antibody responses. Sci Immunol (2017) 2:eaam9169. doi: 10.1126/sciimmunol.aam9169 29196450 PMC7847246

[85] Yu CI Becker C Metang P Marches F Wang Y Toshiyuki H. Human CD141^+^ Dendritic cells induce CD4^+^ T cells to produce type 2 cytokines. J Immunol (2014) 193:4335–43. doi: 10.4049/jimmunol.1401159 PMC4201960 25246496

[86] Bourdely P Anselmi G Vaivode K Ramos RN Missolo-Koussou Y Hidalgo S. Transcriptional and functional analysis of CD1c+ Human dendritic cells identifies a CD163+ Subset priming CD8+CD103+ T cells. Immunity (2020) 53:335–352.e8. doi: 10.1016/j.immuni.2020.06.002 32610077 PMC7445430

[87] Cabeza-Cabrerizo M Cardoso A Minutti CM Pereira da Costa M Reis e Sousa C. Dendritic cells revisited. Annu Rev Immunol (2021) 39:131–66. doi: 10.1146/annurev-immunol-061020-053707 33481643

[88] Bonifaz LC Bonnyay DP Charalambous A Darguste DI Fujii S-I Soares H. *In vivo* targeting of antigens to maturing dendritic cells *via* the DEC-205 receptor improves T cell vaccination. J Exp Med (2004) 199:815–24. doi: 10.1084/jem.20032220 PMC2212731 15024047

[89] Hemmi H Idoyaga J Suda K Suda N Kennedy K Noda M. A new triggering receptor expressed on myeloid cells (Trem) family member, Trem-like 4, binds to dead cells and is a DNAX activation protein 12-linked marker for subsets of mouse macrophages and dendritic cells. J Immunol (2009) 182:1278–86. doi: 10.4049/jimmunol.182.3.1278 PMC2843158 19155473

[90] Hemmi H Zaidi N Wang B Matos I Fiorese C Lubkin A. Treml4, an Ig superfamily member, mediates presentation of several antigens to T cells *in vivo*, including protective immunity to HER2 protein. J Immunol (2012) 188:1147–55. doi: 10.4049/jimmunol.1102541 PMC3262937 22210914

[91] Idoyaga J Fiorese C Zbytnuik L Lubkin A Miller J Malissen B. Specialized role of migratory dendritic cells in peripheral tolerance induction. J Clin Invest (2013) 123:844–54. doi: 10.1172/JCI65260 PMC3561796 23298832

[92] Nedeva C Menassa J Duan M Liu C Doerflinger M Kueh AJ. TREML4 receptor regulates inflammation and innate immune cell death during polymicrobial sepsis. Nat Immunol (2020) 21:1585–96. doi: 10.1038/s41590-020-0789-z 33020659

[93] Fossum E Tesfaye DY Bobic S Gudjonsson A Braathen R Lahoud MH. Targeting antigens to different receptors on conventional type 1 dendritic cells impacts the immune response. J Immunol (2020) 205:661–73. doi: 10.4049/jimmunol.1901119 32591401

[94] Bonifaz L Bonnyay D Mahnke K Rivera M Nussenzweig MC Steinman RM. Efficient targeting of protein antigen to the dendritic cell receptor DEC-205 in the steady state leads to antigen presentation on major histocompatibility complex class I products and peripheral CD8^+^ T cell tolerance. J Exp Med (2002) 196:1627–38. doi: 10.1084/jem.20021598 PMC2196060 12486105

[95] Lahoud MH Ahmet F Kitsoulis S Wan SS Vremec D Lee C-N. Targeting Antigen to Mouse Dendritic Cells *via* Clec9A Induces Potent CD4 T Cell Responses Biased toward a Follicular Helper Phenotype. J Immunol (2011) 187:842–50. doi: 10.4049/jimmunol.1101176 21677141

[96] Masterman K-A Haigh OL Tullett KM Leal-Rojas IM Walpole C Pearson FE. Human CLEC9A antibodies deliver NY-ESO-1 antigen to CD141^+^ dendritic cells to activate naïve and memory NY-ESO-1-specific CD8^+^ T cells. J Immunother Cancer (2020) 8:e000691. doi: 10.1136/jitc-2020-000691 32737142 PMC7394304

[97] Lahoud MH Proietto AI Ahmet F Kitsoulis S Eidsmo L Wu L. The C-type lectin Clec12A present on mouse and human dendritic cells can serve as a target for antigen delivery and enhancement of antibody responses. J Immunol (2009) 182:7587–94. doi: 10.4049/jimmunol.0900464 19494282

[98] Tagliani E Guermonprez P Sepúlveda J López-Bravo M Ardavín C Amigorena S. Selection of an antibody library identifies a pathway to induce immunity by targeting CD36 on steady-state CD8α^+^ Dendritic cells. J Immunol (2008) 180:3201–9. doi: 10.4049/jimmunol.180.5.3201 18292544

[99] Gudjonsson A Lysén A Balan S Sundvold-Gjerstad V Arnold-Schrauf C Richter L. Targeting influenza virus hemagglutinin to Xcr1^+^ Dendritic cells in the absence of receptor-mediated endocytosis enhances protective antibody responses. J Immunol (2017) 198:2785–95. doi: 10.4049/jimmunol.1601881 28228559

[100] Fossum E Grødeland G Terhorst D Tveita AA Vikse E Mjaaland S. Vaccine molecules targeting Xcr1 on cross-presenting DCs induce protective CD8^+^ T-cell responses against influenza virus: New technology. Eur J Immunol (2015) 45:624–35. doi: 10.1002/eji.201445080 25410055

[101] Flinsenberg TWH Compeer EB Koning D Klein M Amelung FJ van Baarle D. Fcγ receptor antigen targeting potentiates cross-presentation by human blood and lymphoid tissue BDCA-3^+^ dendritic cells. Blood (2012) 120:5163–72. doi: 10.1182/blood-2012-06-434498 23093620

[102] Delneste Y Magistrelli G Gauchat J-F Haeuw J-F Aubry J-P Nakamura K. Involvement of LOX-1 in dendritic cell-mediated antigen cross-presentation. Immunity (2002) 17:353–62. doi: 10.1016/S1074-7613(02)00388-6 12354387

[103] Xie J Zhu H Guo L Ruan Y Wang L Sun L. Lectin-like oxidized low-density lipoprotein receptor-1 delivers heat shock protein 60-fused antigen into the MHC class I presentation pathway. J Immunol (2010) 185:2306–13. doi: 10.4049/jimmunol.0903214 20631313

[104] Li D Romain G Flamar A-L Duluc D Dullaers M Li X-H. Targeting self- and foreign antigens to dendritic cells *via* DC-ASGPR generates IL-10–producing suppressive CD4^+^ T cells. J Exp Med (2012) 209:109–21. doi: 10.1084/jem.20110399 PMC3260876 22213806

[105] Lehmann CHK Baranska A Heidkamp GF Heger L Neubert K Lühr JJ. DC subset–specific induction of T cell responses upon antigen uptake *via* Fcγ receptors *in vivo* . J Exp Med (2017) 214:1509–28. doi: 10.1084/jem.20160951 PMC5413326 28389502

[106] Dorner BG Dorner MB Zhou X Opitz C Mora A Güttler S. Selective expression of the chemokine receptor XCR1 on cross-presenting dendritic cells determines cooperation with CD8^+^ T cells. Immunity (2009) 31:823–33. doi: 10.1016/j.immuni.2009.08.027 19913446

[107] Tesfaye DY Bobic S Lysén A Huszthy PC Gudjonsson A Braathen R. Targeting xcr1 on dendritic cells rapidly induce th1-associated immune responses that contribute to protection against influenza infection. Front Immunol (2022) 13:752714. doi: 10.3389/fimmu.2022.752714 35296089 PMC8918470

[108] Tsuji T Matsuzaki J Kelly MP Ramakrishna V Vitale L He L-Z. Antibody-targeted NY-ESO-1 to mannose receptor or DEC-205 *in vitro* elicits dual human CD8^+^ and CD4^+^ T cell responses with broad antigen specificity. J Immunol (2011) 186:1218–27. doi: 10.4049/jimmunol.1000808 21149605

[109] Meixlsperger S Leung CS Rämer PC Pack M Vanoaica LD Breton G. CD141^+^ dendritic cells produce prominent amounts of IFN-α after dsRNA recognition and can be targeted *via* DEC-205 in humanized mice. Blood (2013) 121:5034–44. doi: 10.1182/blood-2012-12-473413 PMC3689250 23482932

[110] Dhodapkar MV Sznol M Zhao B Wang D Carvajal RD Keohan ML. Induction of antigen-specific immunity with a vaccine targeting NY-ESO-1 to the dendritic cell receptor DEC-205. Sci Transl Med (2014) 6:232–52. doi: 10.1126/scitranslmed.3008068 PMC6151129 24739759

[111] Caminschi I Proietto AI Ahmet F Kitsoulis S Shin Teh J Lo JCY. The dendritic cell subtype-restricted C-type lectin Clec9A is a target for vaccine enhancement. Blood (2008) 112:3264–73. doi: 10.1182/blood-2008-05-155176 PMC2569177 18669894

[112] Sancho D Mourão-Sá D Joffre OP Schulz O Rogers NC Pennington DJ. Tumor therapy in mice *via* antigen targeting to a novel, DC-restricted C-type lectin. J Clin Invest (2008) 118:2098–110. doi: 10.1172/JCI34584 PMC2391066 18497879

[113] Canton J Blees H Henry CM Buck MD Schulz O Rogers NC. The receptor DNGR-1 signals for phagosomal rupture to promote cross-presentation of dead-cell-associated antigens. Nat Immunol (2021) 22:140–53. doi: 10.1038/s41590-020-00824-x PMC7116638 33349708

[114] Li J Ahmet F Sullivan LC Brooks AG Kent SJ De Rose R. Antibodies targeting Clec9A promote strong humoral immunity without adjuvant in mice and non-human primates: Immunomodulation. Eur J Immunol (2015) 45:854–64. doi: 10.1002/eji.201445127 25487143

[115] Joffre OP Sancho D Zelenay S Keller AM Reis e Sousa C. Efficient and versatile manipulation of the peripheral CD4^+^ T-cell compartment by antigen targeting to DNGR-1/CLEC9A. Eur J Immunol (2010) 40:1255–65. doi: 10.1002/eji.201040419 PMC3064981 20333625

[116] Kato Y Zaid A Davey GM Mueller SN Nutt SL Zotos D. Targeting antigen to clec9A primes follicular Th cell memory responses capable of robust recall. J Immunol (2015) 195:1006–14. doi: 10.4049/jimmunol.1500767 26101322

[117] Kato Y Steiner TM Park H-Y Hitchcock RO Zaid A Hor JL. Display of native antigen on cDC1 that have spatial access to both T and B cells underlies efficient humoral vaccination. J Immunol (2020) 205:1842–56. doi: 10.4049/jimmunol.2000549 PMC7504891 32839238

[118] Parlato S Romagnoli G Spadaro F Canini I Sirabella P Borghi P. LOX-1 as a natural IFN-α–mediated signal for apoptotic cell uptake and antigen presentation in dendritic cells. Blood (2010) 115:1554–63. doi: 10.1182/blood-2009-07-234468 20009034

[119] Junker F Gordon J Qureshi O. Fc gamma receptors and their role in antigen uptake, presentation, and T cell activation. Front Immunol (2020) 11:1393. doi: 10.3389/fimmu.2020.01393 32719679 PMC7350606

[120] Alcaide EG Krishnarajah S Junker F. Dendritic cell tumor vaccination *via* fc gamma receptor targeting: lessons learned from pre-clinical and translational studies. Vaccines (2021) 9:409. doi: 10.3390/vaccines9040409 33924183 PMC8074394

[121] Bruhns P. Properties of mouse and human IgG receptors and their contribution to disease models. Blood (2012) 119:5640–9. doi: 10.1182/blood-2012-01-380121 22535666

[122] Bruhns P Iannascoli B England P Mancardi DA Fernandez N Jorieux S. Specificity and affinity of human Fcγ receptors and their polymorphic variants for human IgG subclasses. Blood (2009) 113:3716–25. doi: 10.1182/blood-2008-09-179754 19018092

[123] Kroczek AL Hartung E Gurka S Becker M Reeg N Mages HW. Structure-function relationship of XCL1 used for in *vivo* targeting of antigen into XCR1+ Dendritic cells. Front Immunol (2018) 9:2806. doi: 10.3389/fimmu.2018.02806 30619244 PMC6300513

[124] Harshyne LA Watkins SC Gambotto A Barratt-Boyes SM. Dendritic cells acquire antigens from live cells for cross-presentation to CTL. J Immunol (2001) 166:3717–23. doi: 10.4049/jimmunol.166.6.3717 11238612

[125] Dudziak D Kamphorst AO Heidkamp GF Buchholz VR Trumpfheller C Yamazaki S. Differential antigen processing by dendritic cell subsets in vivo. Science (2007) 315:107–11. doi: 10.1126/science.1136080 17204652

[126] Chappell CP Draves KE Giltiay NV Clark EA. Extrafollicular B cell activation by marginal zone dendritic cells drives T cell–dependent antibody responses. J Exp Med (2012) 209:1825–40. doi: 10.1084/jem.20120774 PMC3457737 22966002

[127] Uto T Fukaya T Takagi H Arimura K Nakamura T Kojima N. Clec4A4 is a regulatory receptor for dendritic cells that impairs inflammation and T-cell immunity. Nat Commun (2016) 7:11273. doi: 10.1038/ncomms11273 27068492 PMC4832068

[128] Neubert K Lehmann CHK Heger L Baranska A Staedtler AM Buchholz VR. Antigen delivery to CD11c^+^ CD8^–^ dendritic cells induces protective immune responses against experimental melanoma in mice *in vivo* . J Immunol (2014) 192:5830–8. doi: 10.4049/jimmunol.1300975 24829411

[129] Lysén A Braathen R Gudjonsson A Tesfaye DY Bogen B Fossum E. Dendritic cell targeted Ccl3- and Xcl1-fusion DNA vaccines differ in induced immune responses and optimal delivery site. Sci Rep (2019) 9:1820. doi: 10.1038/s41598-018-38080-7 30755656 PMC6372594

[130] Kamphorst AO Guermonprez P Dudziak D Nussenzweig MC. Route of antigen uptake differentially impacts presentation by dendritic cells and activated monocytes. J Immunol (2010) 185:3426–35. doi: 10.4049/jimmunol.1001205 PMC3013633 20729332

[131] Carter RW Thompson C Reid DM Wong SYC Tough DF. Preferential induction of CD4^+^ T cell responses through *in vivo* targeting of antigen to dendritic cell-associated C-type lectin-1. J Immunol (2006) 177:2276–84. doi: 10.4049/jimmunol.177.4.2276 16887988

[132] Carter RW Thompson C Reid DM Wong SYC Tough DF. Induction of CD8^+^ T cell responses through targeting of antigen to Dectin-2. Cell Immunol (2006) 239:87–91. doi: 10.1016/j.cellimm.2006.05.001 16781694

[133] Corbett AJ Caminschi I McKenzie BS Brady JL Wright MD Mottram PL. Antigen delivery *via* two molecules on the CD8^-^ dendritic cell subset induces humoral immunity in the absence of conventional “danger”. Eur J Immunol (2005) 35:2815–25. doi: 10.1002/eji.200526100 16143986

[134] Schetters STT Kruijssen LJW Crommentuijn MHW Kalay H Ochando J den Haan JMM. Mouse DC-SIGN/CD209a as target for antigen delivery and adaptive immunity. Front Immunol (2018) 9:990. doi: 10.3389/fimmu.2018.00990 29867967 PMC5949514

[135] Papaioannou NE Salei N Rambichler S Ravi K Popovic J Küntzel V. Environmental signals rather than layered ontogeny imprint the function of type 2 conventional dendritic cells in young and adult mice. Nat Commun (2021) 12:464. doi: 10.1038/s41467-020-20659-2 33469015 PMC7815729

[136] Castenmiller C Keumatio-Doungtsop B-C Van Ree R De Jong EC Van Kooyk Y. Tolerogenic immunotherapy: targeting DC surface receptors to induce antigen-specific tolerance. Front Immunol (2021) 12:643240. doi: 10.3389/fimmu.2021.643240 33679806 PMC7933040

[137] Kaifu T Iwakura Y. Dendritic cell immunoreceptor (DCIR): an ITIM-harboring C-type lectin receptor. In: Yamasaki S, editor. C-type lectin receptors in immunity. Tokyo: Springer Japan (2016). p. 101–13. doi: 10.1007/978-4-431-56015-9_7

[138] Kerscher B Willment JA Brown GD. The Dectin-2 family of C-type lectin-like receptors: an update. Int Immunol (2013) 25:271–7. doi: 10.1093/intimm/dxt006 PMC3631001 23606632

[139] Klechevsky E Flamar A-L Cao Y Blanck J-P Liu M O’Bar A. Cross-priming CD8^+^ T cells by targeting antigens to human dendritic cells through DCIR. Blood (2010) 116:1685–97. doi: 10.1182/blood-2010-01-264960 PMC2947393 20530286

[140] Tanriver Y Ratnasothy K Bucy RP Lombardi G Lechler R. Targeting MHC class I monomers to dendritic cells inhibits the indirect pathway of allorecognition and the production of IgG alloantibodies leading to long-term allograft survival. J Immunol (2010) 184:1757–64. doi: 10.4049/jimmunol.0902987 20083658

[141] Tabansky I Keskin DB Watts D Petzold C Funaro M Sands W. Targeting DEC-205–DCIR2^+^ dendritic cells promotes immunological tolerance in proteolipid protein-induced experimental autoimmune encephalomyelitis. Mol Med (2018) 24:17. doi: 10.1186/s10020-018-0017-6 30134798 PMC6016871

[142] Guilliams M Ginhoux F Jakubzick C Naik SH Onai N Schraml BU. Dendritic cells, monocytes and macrophages: a unified nomenclature based on ontogeny. Nat Rev Immunol (2014) 14:571–8. doi: 10.1038/nri3712 PMC4638219 25033907

[143] Xu Y Zhan Y Lew AM Naik SH Kershaw MH. Differential development of murine dendritic cells by GM-CSF versus Flt3 ligand has implications for inflammation and trafficking. J Immunol (2007) 179:7577–84. doi: 10.4049/jimmunol.179.11.7577 18025203

[144] Shibata T Takemura N Motoi Y Goto Y Karuppuchamy T Izawa K. PRAT4A-dependent expression of cell surface TLR5 on neutrophils, classical monocytes and dendritic cells. Int Immunol (2012) 24:613–23. doi: 10.1093/intimm/dxs068 22836022

[145] Reis e Sousa C. Toll-like receptors and dendritic cells: for whom the bug tolls. Semin Immunol (2004) 16:27–34. doi: 10.1016/j.smim.2003.10.004 14751761

[146] Ariizumi K Shen G-L Shikano S Xu S Ritter R Kumamoto T. Identification of a novel, dendritic cell-associated molecule, dectin-1, by subtractive cDNA cloning. J Biol Chem (2000) 275:20157–67. doi: 10.1074/jbc.M909512199 10779524

[147] Lundberg K Rydnert F Greiff L Lindstedt M. Human blood dendritic cell subsets exhibit discriminative pattern recognition receptor profiles. Immunology (2014) 142:279–88. doi: 10.1111/imm.12252 PMC4008235 24444310

[148] Tacken PJ Ginter W Berod L Cruz LJ Joosten B Sparwasser T. Targeting DC-SIGN *via* its neck region leads to prolonged antigen residence in early endosomes, delayed lysosomal degradation, and cross-presentation. Blood (2011) 118:4111–9. doi: 10.1182/blood-2011-04-346957 21860028

[149] Geijtenbeek TBH Torensma R van Vliet SJ van Duijnhoven GCF Adema GJ van Kooyk Y. Identification of DC-SIGN, a novel dendritic cell–specific ICAM-3 receptor that supports primary immune responses. Cell (2000) 100:575–85. doi: 10.1016/S0092-8674(00)80693-5 10721994

[150] Caminschi I Lucas KM O’Keeffe MA Hochrein H Laâbi Y Köntgen F. Molecular cloning of F4/80-like-receptor, a seven-span membrane protein expressed differentially by dendritic cell and monocyte-macrophage subpopulations. J Immunol (2001) 167:3570–6. doi: 10.4049/jimmunol.167.7.3570 11564768

[151] Van Vliet SJ Bay S Vuist IM Kalay H García-Vallejo JJ Leclerc C. MGL signaling augments TLR2-mediated responses for enhanced IL-10 and TNF-α secretion. J Leukocyte Biol (2013) 94:315–23. doi: 10.1189/jlb.1012520 23744646

[152] Ilarregui JM Kooij G Rodríguez E van der Pol SMA Koning N Kalay H. Macrophage galactose-type lectin (MGL) is induced on M2 microglia and participates in the resolution phase of autoimmune neuroinflammation. J Neuroinflammation (2019) 16:130. doi: 10.1186/s12974-019-1522-4 31248427 PMC6598247

[153] Heger L Balk S Lühr JJ Heidkamp GF Lehmann CHK Hatscher L. CLEC10A is a specific marker for human CD1c+ Dendritic cells and enhances their toll-like receptor 7/8-induced cytokine secretion. Front Immunol (2018) 9:744. doi: 10.3389/fimmu.2018.00744 29755453 PMC5934495

[154] Denda-Nagai K Aida S Saba K Suzuki K Moriyama S Oo-puthinan S. Distribution and function of macrophage galactose-type C-type lectin 2 (MGL2/CD301b). J Biol Chem (2010) 285:19193–204. doi: 10.1074/jbc.M110.113613 PMC2885198 20304916

[155] Eggink LL Roby KF Cote R Kenneth Hoober J. An innovative immunotherapeutic strategy for ovarian cancer: CLEC10A and glycomimetic peptides. J Immunother Cancer (2018) 6:28. doi: 10.1186/s40425-018-0339-5 29665849 PMC5905120

[156] Saitoh S-I Abe F Kanno A Tanimura N Mori Saitoh Y Fukui R. TLR7 mediated viral recognition results in focal type I interferon secretion by dendritic cells. Nat Commun (2017) 8:1592. doi: 10.1038/s41467-017-01687-x 29150602 PMC5693993

[157] Benitez-Ribas D Tacken P Punt CJA de Vries IJM Figdor CG. Activation of human plasmacytoid dendritic cells by TLR9 impairs fcγRII-mediated uptake of immune complexes and presentation by MHC class II. J Immunol (2008) 181:5219–24. doi: 10.4049/jimmunol.181.8.5219 18832675

[158] Tel J Sittig SP Blom RAM Cruz LJ Schreibelt G Figdor CG. Targeting uptake receptors on human plasmacytoid dendritic cells triggers antigen cross-presentation and robust type I IFN secretion. J Immunol (2013) 191:5005–12. doi: 10.4049/jimmunol.1300787 24127556

[159] Meyer-Wentrup F Benitez-Ribas D Tacken PJ Punt CJA Figdor CG de Vries IJM. Targeting DCIR on human plasmacytoid dendritic cells results in antigen presentation and inhibits IFN-α production. Blood (2008) 111:4245–53. doi: 10.1182/blood-2007-03-081398 18258799

[160] Ross RL Corinaldesi C Migneco G Carr IM Antanaviciute A Wasson CW. Targeting human plasmacytoid dendritic cells through BDCA2 prevents skin inflammation and fibrosis in a novel xenotransplant mouse model of scleroderma. Ann Rheum Dis (2021) 80:920–9. doi: 10.1136/annrheumdis-2020-218439 PMC8237203 33542104

[161] Sepulveda-Toepfer JA Pichler J Fink K Sevo M Wildburger S Mudde-Boer LC. TLR9-mediated activation of dendritic cells by CD32 targeting for the generation of highly immunostimulatory vaccines. Hum Vaccines Immunotherapeutics (2019) 15:179–88. doi: 10.1080/21645515.2018.1514223 PMC6363151 30156957

[162] Zhang J Raper A Sugita N Hingorani R Salio M Palmowski MJ. Characterization of Siglec-H as a novel endocytic receptor expressed on murine plasmacytoid dendritic cell precursors. Blood (2006) 107:3600–8. doi: 10.1182/blood-2005-09-3842 16397130

[163] Loschko J Heink S Hackl D Dudziak D Reindl W Korn T. Antigen targeting to plasmacytoid dendritic cells *via* siglec-H inhibits Th cell-dependent autoimmunity. J Immunol (2011) 187:6346–56. doi: 10.4049/jimmunol.1102307 22079988

[164] Loschko J Schlitzer A Dudziak D Drexler I Sandholzer N Bourquin C. Antigen delivery to plasmacytoid dendritic cells *via* BST2 induces protective T cell-mediated immunity. J Immunol (2011) 186:6718–25. doi: 10.4049/jimmunol.1004029 21555533

[165] Watarai H Sekine E Inoue S Nakagawa R Kaisho T Taniguchi M. PDC-TREM, a plasmacytoid dendritic cell-specific receptor, is responsible for augmented production of type I interferon. Proc Natl Acad Sci USA (2008) 105:2993–8. doi: 10.1073/pnas.0710351105 PMC2268573 18287072

[166] Le Tortorec A Willey S Neil SJD. Antiviral inhibition of enveloped virus release by tetherin/BST-2: action and counteraction. Viruses (2011) 3:520–40. doi: 10.3390/v3050520 PMC3185764 21994744

[167] Blasius AL Giurisato E Cella M Schreiber RD Shaw AS Colonna M. Bone Marrow Stromal Cell Antigen 2 Is a Specific Marker of Type I IFN-Producing Cells in the Naive Mouse, but a Promiscuous Cell Surface Antigen following IFN Stimulation. J Immunol (2006) 177:3260–5. doi: 10.4049/jimmunol.177.5.3260 16920966

[168] Fu C Peng P Loschko J Feng L Pham P Cui W. Plasmacytoid dendritic cells cross-prime naive CD8 T cells by transferring antigen to conventional dendritic cells through exosomes. Proc Natl Acad Sci USA (2020) 117:23730–41. doi: 10.1073/pnas.2002345117 PMC7519282 32879009

[169] Balan S Saxena M Bhardwaj N. Dendritic cell subsets and locations. Int Rev Cell Mol Biol (2019) 348:1–68. doi: 10.1016/bs.ircmb.2019.07.004 31810551

[170] Persson EK Scott CL McI. Mowat A Agace WW. Dendritic cell subsets in the intestinal lamina propria: Ontogeny and function. Eur J Immunol (2013) 43:3098–107. doi: 10.1002/eji.201343740 PMC3933733 23966272

[171] Stagg AJ. Intestinal dendritic cells in health and gut inflammation. Front Immunol (2018) 9:2883. doi: 10.3389/fimmu.2018.02883 30574151 PMC6291504

[172] Guilliams M Lambrecht BN Hammad H. Division of labor between lung dendritic cells and macrophages in the defense against pulmonary infections. Mucosal Immunol (2013) 6:464–73. doi: 10.1038/mi.2013.14 23549447

[173] Semmrich M Plantinga M Svensson-Frej M Uronen-Hansson H Gustafsson T Mowat AM. Directed antigen targeting in *vivo* identifies a role for CD103^+^ dendritic cells in both tolerogenic and immunogenic T-cell responses. Mucosal Immunol (2012) 5:150–60. doi: 10.1038/mi.2011.61 PMC3282433 22166938

[174] Wang D Yuan R Feng Y El-Asady R Farber DL Gress RE. Regulation of CD103 expression by CD8^+^ T cells responding to renal allografts. J Immunol (2004) 172:214–21. doi: 10.4049/jimmunol.172.1.214 14688328

[175] Zhang Y Chen G Liu Z Tian S Zhang J Carey CD. Genetic vaccines to potentiate the effective CD103^+^ Dendritic cell–mediated cross-priming of antitumor immunity. J Immunol (2015) 194:5937–47. doi: 10.4049/jimmunol.1500089 PMC4458448 25972487

[176] Figdor CG van Kooyk Y Adema GJ. C-type lectin receptors on dendritic cells and langerhans cells. Nat Rev Immunol (2002) 2:77–84. doi: 10.1038/nri723 11910898

[177] ROmani N Clausen BE Stoitzner P. Langerhans cells and more: langerin-expressing dendritic cell subsets in the skin. Immunol Rev (2010) 234:120–41. doi: 10.1111/j.0105-2896.2009.00886.x PMC2907488 20193016

[178] Merad M Ginhoux F Collin M. Origin, homeostasis and function of Langerhans cells and other langerin-expressing dendritic cells. Nat Rev Immunol (2008) 8:935–47. doi: 10.1038/nri2455 19029989

[179] West HC Bennett CL. Redefining the role of langerhans cells as immune regulators within the skin. Front Immunol (2018) 8:1941. doi: 10.3389/fimmu.2017.01941 29379502 PMC5770803

[180] Kaplan DH. Ontogeny and function of murine epidermal Langerhans cells. Nat Immunol (2017) 18:1068–75. doi: 10.1038/ni.3815 PMC6422157 28926543

[181] Satpathy AT Wu X Albring JC Murphy KM. Re(de)fining the dendritic cell lineage. Nat Immunol (2012) 13:1145–54. doi: 10.1038/ni.2467 PMC3644874 23160217

[182] Romani N Thurnher M Idoyaga J Steinman RM Flacher V. Targeting of antigens to skin dendritic cells: possibilities to enhance vaccine efficacy. Immunol Cell Biol (2010) 88:424–30. doi: 10.1038/icb.2010.39 PMC2907485 20368713

[183] Li R-JE Hogervorst TP Achilli S Bruijns SCM Spiekstra S Vivès C. Targeting of the C-type lectin receptor langerin using bifunctional mannosylated antigens. Front Cell Dev Biol (2020) 8:556. doi: 10.3389/fcell.2020.00556 32760719 PMC7371993

[184] Bellmann L Strandt H Zelle-Rieser C Ortner D Tripp CH Schmid S. Targeted delivery of a vaccine protein to Langerhans cells in the human skin *via* the C-type lectin receptor Langerin. Eur J Immunol (2022) 52:1829–41. doi: 10.1002/eji.202149670 PMC9788233 34932821

[185] Fukunaga A Khaskhely NM Sreevidya CS Byrne SN Ullrich SE. Dermal dendritic cells, and not langerhans cells, play an essential role in inducing an immune response. J Immunol (2008) 180:3057–64. doi: 10.4049/jimmunol.180.5.3057 PMC2408377 18292528

[186] Mair F Liechti T. Comprehensive phenotyping of human dendritic cells and monocytes. Cytometry (2021) 99:231–42. doi: 10.1002/cyto.a.24269 33200508

[187] Dutertre C-A Becht E Irac SE Khalilnezhad A Narang V Khalilnezhad S. Single-cell analysis of human mononuclear phagocytes reveals subset-defining markers and identifies circulating inflammatory dendritic cells. Immunity (2019) 51:573–589.e8. doi: 10.1016/j.immuni.2019.08.008 31474513

[188] Leach SM Gibbings SL Tewari AD Atif SM Vestal B Danhorn T. Human and mouse transcriptome profiling identifies cross-species homology in pulmonary and lymph node mononuclear phagocytes. Cell Rep (2020) 33:108337. doi: 10.1016/j.celrep.2020.108337 33147458 PMC7673261

[189] Nakano H Moran TP Nakano K Gerrish KE Bortner CD Cook DN. Complement receptor C5aR1/CD88 and dipeptidyl peptidase-4/CD26 define distinct hematopoietic lineages of dendritic cells. J Immunol (2015) 194:3808–19. doi: 10.4049/jimmunol.1402195 PMC4390500 25769922

[190] Huang M-N Nicholson LT Batich KA Swartz AM Kopin D Wellford S. Antigen-loaded monocyte administration induces potent therapeutic antitumor T cell responses. J Clin Invest (2020) 130:774–88. doi: 10.1172/JCI128267 PMC6994156 31661470

[191] Lee SH Park OK Kim J Shin K Pack CG Kim K. Deep tumor penetration of drug-loaded nanoparticles by click reaction-assisted immune cell targeting strategy. J Am Chem Soc (2019) 141:13829–40. doi: 10.1021/jacs.9b04621 31382746

[192] Veiga N Goldsmith M Granot Y Rosenblum D Dammes N Kedmi R. Cell specific delivery of modified mRNA expressing therapeutic proteins to leukocytes. Nat Commun (2018) 9:4493. doi: 10.1038/s41467-018-06936-1 30374059 PMC6206083

[193] Lin Y Slight SR Khader SA. Th17 cytokines and vaccine-induced immunity. Semin Immunopathol (2010) 32:79–90. doi: 10.1007/s00281-009-0191-2 20112107 PMC2855296

[194] Reith W LeibundGut-Landmann S Waldburger J-M. Regulation of MHC class II gene expression by the class II transactivator. Nat Rev Immunol (2005) 5:793–806. doi: 10.1038/nri1708 16200082

[195] Buxadé M Huerga Encabo H Riera-Borrull M Quintana-Gallardo L López-Cotarelo P Tellechea M. Macrophage-specific MHCII expression is regulated by a remote Ciita enhancer controlled by NFAT5. J Exp Med (2018) 215:2901–18. doi: 10.1084/jem.20180314 PMC6219740 30327417

[196] Muntjewerff EM Meesters LD van den Bogaart G. Antigen cross-Presentation by macrophages. Front Immunol (2020) 11:1276. doi: 10.3389/fimmu.2020.01276 32733446 PMC7360722

[197] Murray PJ Wynn TA. Protective and pathogenic functions of macrophage subsets. Nat Rev Immunol (2011) 11:723–37. doi: 10.1038/nri3073 PMC3422549 21997792

[198] Chen B Li R Kubota A Alex L Frangogiannis NG. Identification of macrophages in normal and injured mouse tissues using reporter lines and antibodies. Sci Rep (2022) 12:4542. doi: 10.1038/s41598-022-08278-x 35296717 PMC8927419

[199] Duan Z Luo Y. Targeting macrophages in cancer immunotherapy. Sig Transduct Target Ther (2021) 6:127. doi: 10.1038/s41392-021-00506-6 PMC7994399 33767177

[200] Qian B-Z Pollard JW. Macrophage diversity enhances tumor progression and metastasis. Cell (2010) 141:39–51. doi: 10.1016/j.cell.2010.03.014 20371344 PMC4994190

[201] Davies LC Jenkins SJ Allen JE Taylor PR. Tissue-resident macrophages. Nat Immunol (2013) 14:986–95. doi: 10.1038/ni.2705 PMC4045180 24048120

[202] Geissmann F Manz MG Jung S Sieweke MH Merad M Ley K. Development of monocytes, macrophages, and dendritic cells. Science (2010) 327:656–61. doi: 10.1126/science.1178331 PMC2887389 20133564

[203] Ngambenjawong C Gustafson HH Pun SH. Progress in tumor-associated macrophage (TAM)-targeted therapeutics. Adv Drug Deliv Rev (2017) 114:206–21. doi: 10.1016/j.addr.2017.04.010 PMC5581987 28449873

[204] Colino CI Lanao JM Gutierrez-Millan C. Targeting of hepatic macrophages by therapeutic nanoparticles. Front Immunol (2020) 11:218. doi: 10.3389/fimmu.2020.00218 32194546 PMC7065596

[205] Weiss AM Hossainy S Rowan SJ Hubbell JA Esser-Kahn AP. Immunostimulatory polymers as adjuvants, immunotherapies, and delivery systems. Macromolecules (2022) 55:6913–37. doi: 10.1021/acs.macromol.2c00854 PMC9404695 36034324

[206] Laroui H Viennois E Xiao B Canup BSB Geem D Denning TL. Fab’-bearing siRNA TNFα-loaded nanoparticles targeted to colonic macrophages offer an effective therapy for experimental colitis. J Controlled Release (2014) 186:41–53. doi: 10.1016/j.jconrel.2014.04.046 PMC4100604 24810114

[207] Chávez-Galán L Olleros ML Vesin D Garcia I. Much More than M1 and M2 Macrophages, There are also CD169^+^ and TCR^+^ Macrophages. Front Immunol (2015) 6:263. doi: 10.3389/fimmu.2015.00263 26074923 PMC4443739

[208] Zhang C Yang M Ericsson AC. Function of macrophages in disease: current understanding on molecular mechanisms. Front Immunol (2021) 12:620510. doi: 10.3389/fimmu.2021.620510 33763066 PMC7982479

[209] Hu G Guo M Xu J Wu F Fan J Huang Q. Nanoparticles targeting macrophages as potential clinical therapeutic agents against cancer and inflammation. Front Immunol (2019) 10:1998. doi: 10.3389/fimmu.2019.01998 31497026 PMC6712945

[210] Cieslewicz M Tang J Yu JL Cao H Zavaljevski M Motoyama K. Targeted delivery of proapoptotic peptides to tumor-associated macrophages improves survival. Proc Natl Acad Sci USA (2013) 110:15919–24. doi: 10.1073/pnas.1312197110 PMC3791765 24046373

[211] Jain S Tran T-H Amiji M. Macrophage repolarization with targeted alginate nanoparticles containing IL-10 plasmid DNA for the treatment of experimental arthritis. Biomaterials (2015) 61:162–77. doi: 10.1016/j.biomaterials.2015.05.028 PMC4464978 26004232

[212] Bar-Shavit Z Terry S Blumberg S Goldman R. Neurotensin — macrophage interaction: Specific binding and augmentation of phagocytosis. Neuropeptides (1982) 2:325–35. doi: 10.1016/0143-4179(82)90070-1

[213] Farajzadeh R Zarghami N Serati-Nouri H Momeni-Javid Z Farajzadeh T Jalilzadeh-Tabrizi S. Macrophage repolarization using CD44-targeting hyaluronic acid–polylactide nanoparticles containing curcumin. Artif Cells Nanomedicine Biotechnol (2017) 46(8):2013–21. doi: 10.1080/21691401.2017.1408116 29183161

[214] Tran T-H Rastogi R Shelke J Amiji MM. Modulation of macrophage functional polarity towards anti-inflammatory phenotype with plasmid DNA delivery in CD44 targeting hyaluronic acid nanoparticles. Sci Rep (2015) 5:16632. doi: 10.1038/srep16632 26577684 PMC4649614

[215] Alvarado-Vazquez PA Bernal L Paige CA Grosick RL Moracho Vilrriales C Ferreira DW. Macrophage-specific nanotechnology-driven CD163 overexpression in human macrophages results in an M2 phenotype under inflammatory conditions. Immunobiology (2017) 222:900–12. doi: 10.1016/j.imbio.2017.05.011 PMC5718187 28545809

[216] Zhu S Niu M O’Mary H Cui Z. Targeting of tumor-associated macrophages made possible by PEG-sheddable, mannose-modified nanoparticles. Mol Pharmaceutics. (2013) 10:3525–30. doi: 10.1021/mp400216r PMC3946577 23901887

[217] Aouadi M Tencerova M Vangala P Yawe JC Nicoloro SM Amano SU. Gene silencing in adipose tissue macrophages regulates whole-body metabolism in obese mice. Proc Natl Acad Sci USA (2013) 110:8278–83. doi: 10.1073/pnas.1300492110 PMC3657808 23630254

[218] Aouadi M Tesz GJ Nicoloro SM Wang M Chouinard M Soto E. Orally delivered siRNA targeting macrophage Map4k4 suppresses systemic inflammation. Nature (2009) 458:1180–4. doi: 10.1038/nature07774 PMC2879154 19407801

[219] Ngambenjawong C Cieslewicz M Schellinger JG Pun SH. Synthesis and evaluation of multivalent M2pep peptides for targeting alternatively activated M2 macrophages. J Controlled Release (2016) 224:103–11. doi: 10.1016/j.jconrel.2015.12.057 PMC4747818 26772876

[220] Lee C Bae S-JS Joo H Bae H. Melittin suppresses tumor progression by regulating tumor-associated macrophages in a Lewis lung carcinoma mouse model. Oncotarget (2017) 8:54951–65. doi: 10.18632/oncotarget.18627 PMC5589633 28903394

[221] Lee C Jeong H Bae Y Shin K Kang S Kim H. Targeting of M2-like tumor-associated macrophages with a melittin-based pro-apoptotic peptide. J immunotherapy Cancer (2019) 7:147. doi: 10.1186/s40425-019-0610-4 PMC6555931 31174610

[222] Puig-Kröger A Sierra-Filardi E Domínguez-Soto A Samaniego R Corcuera MT Gómez-Aguado F. Folate receptor β Is expressed by tumor-associated macrophages and constitutes a marker for M2 anti-inflammatory/regulatory macrophages. Cancer Res (2009) 69:9395–403. doi: 10.1158/0008-5472.CAN-09-2050 19951991

[223] Hattori Y Yamashita J Sakaida C Kawano K Yonemochi E. Evaluation of antitumor effect of zoledronic acid entrapped in folate-linked liposome for targeting to tumor-associated macrophages. J Liposome Res (2015) 25:131–40. doi: 10.3109/08982104.2014.954128 25203609

[224] Tie Y Zheng H He Z Yang J Shao B Liu L. Targeting folate receptor β positive tumor-associated macrophages in lung cancer with a folate-modified liposomal complex. Sig Transduct Target Ther (2020) 5:6. doi: 10.1038/s41392-020-0115-0 PMC6976681 32296026

[225] Nagai T Tanaka M Tsuneyoshi Y Xu B Michie SA Hasui K. Targeting tumor-associated macrophages in an experimental glioma model with a recombinant immunotoxin to folate receptor β. Cancer Immunol Immunother (2009) 58:1577–86. doi: 10.1007/s00262-009-0667-x PMC11030051 19238383

[226] Crocker PR Gordon S. Properties and distribution of a lectin-like hemagglutinin differentially expressed by murine stromal tissue macrophages. J Exp Med (1986) 164:1862–75. doi: 10.1084/jem.164.6.1862 PMC2188478 3783087

[227] Phan TG Grigorova I Okada T Cyster JG. Subcapsular encounter and complement-dependent transport of immune complexes by lymph node B cells. Nat Immunol (2007) 8:992–1000. doi: 10.1038/ni1494 17660822

[228] Junt T Moseman EA Iannacone M Massberg S Lang PA Boes M. Subcapsular sinus macrophages in lymph nodes clear lymph-borne viruses and present them to antiviral B cells. Nature (2007) 450:110–4. doi: 10.1038/nature06287 17934446

[229] van Dinther D Veninga H Iborra S Borg EGF Hoogterp L Olesek K. Functional CD169 on macrophages mediates interaction with dendritic cells for CD8^+^ T cell cross-priming. Cell Rep (2018) 22:1484–95. doi: 10.1016/j.celrep.2018.01.021 29425504

[230] van Dinther D Veninga H Revet M Hoogterp L Olesek K Grabowska J. Comparison of protein and peptide targeting for the development of a CD169-based vaccination strategy against melanoma. Front Immunol (2018) 9:1997. doi: 10.3389/fimmu.2018.01997 30237798 PMC6135888

[231] Nijen Twilhaar MK Czentner L Grabowska J Affandi AJ Lau CYJ Olesek K. Optimization of liposomes for antigen targeting to splenic CD169^+^ Macrophages. Pharmaceutics (2020) 12:1138. doi: 10.3390/pharmaceutics12121138 33255564 PMC7760819

[232] Kawasaki N Vela JL Nycholat CM Rademacher C Khurana A van Rooijen N. Targeted delivery of lipid antigen to macrophages *via* the CD169/sialoadhesin endocytic pathway induces robust invariant natural killer T cell activation. Proc Natl Acad Sci USA (2013) 110:7826–31. doi: 10.1073/pnas.1219888110 PMC3651435 23610394

[233] Edgar LJ Kawasaki N Nycholat CM Paulson JC. Targeted delivery of antigen to activated CD169^+^ Macrophages induces bias for expansion of CD8^+^ T cells. Cell Chem Biol (2019) 26:131–136.e4. doi: 10.1016/j.chembiol.2018.10.006 30393066 PMC6338492

[234] Lisk C Yuen R Kuniholm J Antos D Reiser ML Wetzler LM. CD169^+^ Subcapsular macrophage role in antigen adjuvant activity. Front Immunol (2021) 12:624197. doi: 10.3389/fimmu.2021.624197 33815376 PMC8012505

[235] Wen T Rothenberg ME. The regulatory function of eosinophils. Microbiol Spectr (2016) 4. doi: 10.1128/microbiolspec.MCHD-0020-2015 PMC5088784 27780017

[236] Maddur MS Kaveri SV Bayry J. Basophils as antigen presenting cells. Trends Immunol (2010) 31:45–8. doi: 10.1016/j.it.2009.12.004 20060781

[237] Galli SJ Gaudenzio N. Human mast cells as antigen-presenting cells: When is this role important *in vivo* ? J Allergy Clin Immunol (2018) 141:92–3. doi: 10.1016/j.jaci.2017.05.029 28624609

[238] Kambayashi T Laufer TM. Atypical MHC class II-expressing antigen-presenting cells: can anything replace a dendritic cell? Nat Rev Immunol (2014) 14:719–30. doi: 10.1038/nri3754 25324123

[239] Abi Abdallah DS Egan CE Butcher BA Denkers EY. Mouse neutrophils are professional antigen-presenting cells programmed to instruct Th1 and Th17 T-cell differentiation. Int Immunol (2011) 23:317–26. doi: 10.1093/intimm/dxr007 PMC3082529 21422151

[240] Trentini MM de Oliveira FM Kipnis A Junqueira-Kipnis AP. The Role of Neutrophils in the Induction of Specific Th1 and Th17 during Vaccination against Tuberculosis. Front Microbiol (2016) 7:898. doi: 10.3389/fmicb.2016.00898 27375607 PMC4901074

[241] Bartneck M Wang J. Therapeutic targeting of neutrophil granulocytes in inflammatory liver disease. Front Immunol (2019) 10:2257. doi: 10.3389/fimmu.2019.02257 31616430 PMC6764082

[242] Filep JG. Targeting neutrophils for promoting the resolution of inflammation. Front Immunol (2022) 13:866747. doi: 10.3389/fimmu.2022.866747 35371088 PMC8966391

[243] Wang Z Li J Cho J Malik AB. Prevention of vascular inflammation by nanoparticle targeting of adherent neutrophils. Nat Nanotech. (2014) 9:204–10. doi: 10.1038/nnano.2014.17 PMC4100792 24561355

[244] Zhang CY Dong X Gao J Lin W Liu Z Wang Z. Nanoparticle-induced neutrophil apoptosis increases survival in sepsis and alleviates neurological damage in stroke. Sci Adv (2019) 5:eaax7964. doi: 10.1126/sciadv.aax7964 31723603 PMC6834394

[245] Vij N Min T Bodas M Gorde A Roy I. Neutrophil targeted nano-drug delivery system for chronic obstructive lung diseases. Nanomedicine: Nanotechnology Biol Med (2016) 12:2415–27. doi: 10.1016/j.nano.2016.06.008 27381067

[246] Cruz MA Bohinc D Andraska EA Alvikas J Raghunathan S Masters NA. Nanomedicine platform for targeting activated neutrophils and neutrophil–platelet complexes using an α1-antitrypsin-derived peptide motif. Nat Nanotechnol (2022) 17:1004–14. doi: 10.1038/s41565-022-01161-w PMC9909445 35851383

[247] Forsyth S Horvath A Coughlin P. A review and comparison of the murine α1-antitrypsin and α1-antichymotrypsin multigene clusters with the human clade A serpins. Genomics (2003) 81:336–45. doi: 10.1016/S0888-7543(02)00041-1 12659817

[248] Völs S Kaisar-Iluz N Shaul ME Ryvkin A Ashkenazy H Yehuda A. Targeted nanoparticles modify neutrophil function *in vivo* . Front Immunol (2022) 13:1003871. doi: 10.3389/fimmu.2022.1003871 36275643 PMC9580275

[249] dos Santos Dias L Silva LBR Nosanchuk JD Taborda CP. Neutrophil cells are essential for the efficacy of a therapeutic vaccine against paracoccidioidomycosis. J Fungi (Basel). (2021) 7:416. doi: 10.3390/jof7060416 34073466 PMC8226764

[250] Calabro S Tortoli M Baudner BC Pacitto A Cortese M O’Hagan DT. Vaccine adjuvants alum and MF59 induce rapid recruitment of neutrophils and monocytes that participate in antigen transport to draining lymph nodes. Vaccine (2011) 29:1812–23. doi: 10.1016/j.vaccine.2010.12.090 21215831

[251] Hogan SP Rosenberg HF Moqbel R Phipps S Foster PS Lacy P. Eosinophils: biological properties and role in health and disease. Clin Exp Allergy (2008) 38:709–50. doi: 10.1111/j.1365-2222.2008.02958.x 18384431

[252] Arinobu Y Iwasaki H Gurish MF Mizuno S Shigematsu H Ozawa H. Developmental checkpoints of the basophil/mast cell lineages in adult murine hematopoiesis. Proc Natl Acad Sci USA (2005) 102:18105–10. doi: 10.1073/pnas.0509148102 PMC1312421 16330751

[253] Piliponsky AM Shubin NJ Lahiri AK Truong P Clauson M Niino K. Basophil-derived tumor necrosis factor can enhance survival in a sepsis model in mice. Nat Immunol (2019) 20:129–40. doi: 10.1038/s41590-018-0288-7 PMC6352314 30664762

[254] Dellon ES Peterson KA Murray JA Falk GW Gonsalves N Chehade M. Anti–siglec-8 antibody for eosinophilic gastritis and duodenitis. N Engl J Med (2020) 383:1624–34. doi: 10.1056/NEJMoa2012047 PMC7600443 33085861

[255] Nycholat CM Duan S Knuplez E Worth C Elich M Yao A. A sulfonamide sialoside analogue for targeting siglec-8 and -F on immune cells. J Am Chem Soc (2019) 141:14032–7. doi: 10.1021/jacs.9b05769 PMC6861165 31460762

[256] Kiwamoto T Kawasaki N Paulson JC Bochner BS. Siglec-8 as a drugable target to treat eosinophil and mast cell-associated conditions. Pharmacol Ther (2012) 135:327–36. doi: 10.1016/j.pharmthera.2012.06.005 PMC3587973 22749793

[257] Rillahan CD Macauley MS Schwartz E He Y McBride R Arlian BM. Disubstituted sialic acid ligands targeting siglecs CD33 and CD22 associated with myeloid leukaemias and B cell lymphomas. Chem Sci (2014) 5:2398. doi: 10.1039/c4sc00451e 24921038 PMC4048721

[258] Duan S Koziol-White CJ Jester WF Nycholat CM Macauley MS Panettieri RA. CD33 recruitment inhibits IgE-mediated anaphylaxis and desensitizes mast cells to allergen. J Clin Invest (2019) 129:1387–401. doi: 10.1172/JCI125456 PMC6391081 30645205

[259] Gibbs BF Yasinska IM Calzolai L Gilliland D Sumbayev VV. Highly specific targeting of human leukocytes using gold nanoparticle-based biologically active conjugates. J Biomed Nanotechnology (2014) 10:1259–66. doi: 10.1166/jbn.2014.1807 24804546

[260] Yasinska IM Calzolai L Raap U Hussain R Siligardi G Sumbayev VV. Targeting of basophil and mast cell pro-allergic reactivity using functionalised gold nanoparticles. Front Pharmacol (2019) 10:333. doi: 10.3389/fphar.2019.00333 30984005 PMC6449467

[261] Irvine DJ Read BJ. Shaping humoral immunity to vaccines through antigen-displaying nanoparticles. Curr Opin Immunol (2020) 65:1–6. doi: 10.1016/j.coi.2020.01.007 32200132 PMC7501207

[262] Jiang H Wang Q Sun X. Lymph node targeting strategies to improve vaccination efficacy. J Controlled Release (2017) 267:47–56. doi: 10.1016/j.jconrel.2017.08.009 28818619

[263] Abdallah M Müllertz OO Styles IK Mörsdorf A Quinn JF Whittaker MR. Lymphatic targeting by albumin-hitchhiking: Applications and optimisation. J Controlled Release (2020) 327:117–28. doi: 10.1016/j.jconrel.2020.07.046 32771478

[264] Reddy ST Swartz MA Hubbell JA. Targeting dendritic cells with biomaterials: developing the next generation of vaccines. Trends Immunol (2006) 27:573–9. doi: 10.1016/j.it.2006.10.005 17049307

[265] McCright J Naiknavare R Yarmovsky J Maisel K. Targeting lymphatics for nanoparticle drug delivery. Front Pharmacol (2022) 13:887402. doi: 10.3389/fphar.2022.887402 35721179 PMC9203826

[266] Swartz M. The physiology of the lymphatic system. Adv Drug Deliv Rev (2001) 50:3–20. doi: 10.1016/S0169-409X(01)00150-8 11489331

[267] Singh A. Eliciting B cell immunity against infectious diseases using nanovaccines. Nat Nanotechnol. (2021) 16:16–24. doi: 10.1038/s41565-020-00790-3 33199883 PMC7855692

[268] Shen L Tenzer S Storck W Hobernik D Raker VK Fischer K. Protein corona–mediated targeting of nanocarriers to B cells allows redirection of allergic immune responses. J Allergy Clin Immunol (2018) 142:1558–70. doi: 10.1016/j.jaci.2017.08.049 29382591

[269] Shimizu T Ishida T Kiwada H. Transport of PEGylated liposomes from the splenic marginal zone to the follicle in the induction phase of the accelerated blood clearance phenomenon. Immunobiology (2013) 218:725–32. doi: 10.1016/j.imbio.2012.08.274 22995937

[270] Shimizu T Mima Y Hashimoto Y Ukawa M Ando H Kiwada H. Anti-PEG IgM and complement system are required for the association of second doses of PEGylated liposomes with splenic marginal zone B cells. Immunobiology (2015) 220:1151–60. doi: 10.1016/j.imbio.2015.06.005 26095176

[271] Shimizu T Abu Lila AS Kawaguchi Y Shimazaki Y Watanabe Y Mima Y. A novel platform for cancer vaccines: antigen-selective delivery to splenic marginal zone B cells *via* repeated injections of PEGylated liposomes. J Immunol (2018) 201:2969–76. doi: 10.4049/jimmunol.1701351 30333124

[272] Shimizu T Awata M Abu Lila AS Yoshioka C Kawaguchi Y Ando H. Complement activation induced by PEG enhances humoral immune responses against antigens encapsulated in PEG-modified liposomes. J Controlled Release. (2021) 329:1046–53. doi: 10.1016/j.jconrel.2020.10.033 33080272

[273] Temchura VV Kozlova D Sokolova V Überla K Epple M. Targeting and activation of antigen-specific B-cells by calcium phosphate nanoparticles loaded with protein antigen. Biomaterials (2014) 35:6098–105. doi: 10.1016/j.biomaterials.2014.04.010 24776487

[274] Zilker C Kozlova D Sokolova V Yan H Epple M Überla K. Nanoparticle-based B-cell targeting vaccines: Tailoring of humoral immune responses by functionalization with different TLR-ligands. Nanomedicine: Nanotechnology Biol Med (2017) 13:173–82. doi: 10.1016/j.nano.2016.08.028 27593489

[275] Moyer TJ Kato Y Abraham W Chang JYH Kulp DW Watson N. Engineered immunogen binding to alum adjuvant enhances humoral immunity. Nat Med (2020) 26:430–40. doi: 10.1038/s41591-020-0753-3 PMC7069805 32066977

[276] Carter RH Fearon DT. CD19: lowering the threshold for antigen receptor stimulation of B lymphocytes. Science (1992) 256:105–7. doi: 10.1126/science.1373518 1373518

[277] Fearon DT Carroll MC Carroll MC. Regulation of B lymphocyte responses to foreign and self-antigens by the CD19/CD21 complex. Annu Rev Immunol (2000) 18:393–422. doi: 10.1146/annurev.immunol.18.1.393 10837064

[278] Yan J Wolff MJ Unternaehrer J Mellman I Mamula MJ. Targeting antigen to CD19 on B cells efficiently activates T cells. Int Immunol (2005) 17:869–77. doi: 10.1093/intimm/dxh266 15967786

[279] Ma Y Xiang D Sun J Ding C Liu M Hu X. Targeting of antigens to B lymphocytes *via* CD19 as a means for tumor vaccine development. J Immunol (2013) 190:5588–99. doi: 10.4049/jimmunol.1203216 PMC3660458 23630363

[280] Ding C Wang L Marroquin J Yan J. Targeting of antigens to B cells augments antigen-specific T-cell responses and breaks immune tolerance to tumor-associated antigen MUC1. Blood (2008) 112:2817–25. doi: 10.1182/blood-2008-05-157396 PMC2556617 18669871

[281] Andersen TK Huszthy PC Gopalakrishnan RP Jacobsen JT Fauskanger M Tveita AA. Enhanced germinal center reaction by targeting vaccine antigen to major histocompatibility complex class II molecules. NPJ Vaccines (2019) 4:9. doi: 10.1038/s41541-019-0101-0 30775000 PMC6370881

[282] Hinke DM Andersen TK Gopalakrishnan RP Skullerud LM Werninghaus IC Grødeland G. Antigen bivalency of antigen-presenting cell-targeted vaccines increases B cell responses. Cell Rep (2022) 39:110901. doi: 10.1016/j.celrep.2022.110901 35649357

[283] van Nierop K. Human follicular dendritic cells: function, origin and development. Semin Immunol (2002) 14:251–7. doi: 10.1016/S1044-5323(02)00057-X 12163300

[284] Kranich J Krautler NJ. How follicular dendritic cells shape the B-cell antigenome. Front Immunol (2016) 7:225. doi: 10.3389/fimmu.2016.00225 27446069 PMC4914831

[285] Heesters BA Chatterjee P Kim Y-A Gonzalez SF Kuligowski MP Kirchhausen T. Endocytosis and recycling of immune complexes by follicular dendritic cells enhances B cell antigen binding and activation. Immunity (2013) 38:1164–75. doi: 10.1016/j.immuni.2013.02.023 PMC3773956 23770227

[286] Heesters BA Myers RC Carroll MC. Follicular dendritic cells: dynamic antigen libraries. Nat Rev Immunol (2014) 14:495–504. doi: 10.1038/nri3689 24948364

[287] Zhang Y-N Lazarovits J Poon W Ouyang B Nguyen LNM Kingston BR. Nanoparticle size influences antigen retention and presentation in lymph node follicles for humoral immunity. Nano Lett (2019) 19:7226–35. doi: 10.1021/acs.nanolett.9b02834 31508968

[288] Mattsson J Yrlid U Stensson A Schön K Karlsson MCI Ravetch JV. Complement activation and complement receptors on follicular dendritic cells are critical for the function of a targeted adjuvant. J Immunol (2011) 187:3641–52. doi: 10.4049/jimmunol.1101107 21880985

[289] Schussek S Bernasconi V Mattsson J Wenzel UA Strömberg A Gribonika I. The CTA1-DD adjuvant strongly potentiates follicular dendritic cell function and germinal center formation, which results in improved neonatal immunization. Mucosal Immunol (2020) 13:545–57. doi: 10.1038/s41385-020-0253-2 PMC7223721 31959882

[290] Aung A Cui A Maiorino L Amini AP Gregory JR BuKenya M. Low protease activity in B cell follicles promotes retention of intact antigens after immunization. Science (2023) 379:eabn8934. doi: 10.1126/science.abn8934 36701450 PMC10041875

[291] Tamburini BA Burchill MA Kedl RM. Antigen capture and archiving by lymphatic endothelial cells following vaccination or viral infection. Nat Commun (2014) 5:3989. doi: 10.1038/ncomms4989 24905362 PMC4073648

[292] Kedl RM Lindsay RS Finlon JM Lucas ED Friedman RS Tamburini BAJ. Migratory dendritic cells acquire and present lymphatic endothelial cell-archived antigens during lymph node contraction. Nat Commun (2017) 8:2034. doi: 10.1038/s41467-017-02247-z 29229919 PMC5725486

[293] Walsh SM Sheridan RM Lucas ED Doan TA Ware BC Schafer J. Molecular tracking devices quantify antigen distribution and archiving in the murine lymph node. eLife (2021) 10:e62781. doi: 10.7554/eLife.62781 33843587 PMC8116055

[294] Vokali E Yu SS Hirosue S Rinçon-Restrepo M Duraes FV Scherer S. Lymphatic endothelial cells prime naïve CD8^+^ T cells into memory cells under steady-state conditions. Nat Commun (2020) 11:538. doi: 10.1038/s41467-019-14127-9 31988323 PMC6985113

[295] Tewalt EF Cohen JN Rouhani SJ Guidi CJ Qiao H Fahl SP. Lymphatic endothelial cells induce tolerance *via* PD-L1 and lack of costimulation leading to high-level PD-1 expression on CD8 T cells. Blood (2012) 120:4772–82. doi: 10.1182/blood-2012-04-427013 PMC3520619 22993390

[296] Rouhani SJ Eccles JD Riccardi P Peske JD Tewalt EF Cohen JN. Roles of lymphatic endothelial cells expressing peripheral tissue antigens in CD4 T-cell tolerance induction. Nat Commun (2015) 6:6771. doi: 10.1038/ncomms7771 25857745 PMC4403767

[297] Dubrot J Duraes FV Potin L Capotosti F Brighouse D Suter T. Lymph node stromal cells acquire peptide–MHCII complexes from dendritic cells and induce antigen-specific CD4^+^ T cell tolerance. J Exp Med (2014) 211:1153–66. doi: 10.1084/jem.20132000 PMC4042642 24842370

[298] Humbert M Hugues S Dubrot J. Shaping of peripheral T cell responses by lymphatic endothelial cells. Front Immunol (2017) 7:684. doi: 10.3389/fimmu.2016.00684 28127298 PMC5226940

[299] Krishnamurty AT Turley SJ. Lymph node stromal cells: cartographers of the immune system. Nat Immunol (2020) 21:369–80. doi: 10.1038/s41590-020-0635-3 32205888

[300] Grasso C Pierie C Mebius RE van Baarsen LGM. Lymph node stromal cells: subsets and functions in health and disease. Trends Immunol (2021) 42:920–36. doi: 10.1016/j.it.2021.08.009 34521601

[301] Guo Q Xu Ke Ren Ke Sun W Liu Yi. Mouse lymphatic endothelial cell targeted probes: anti-LYVE-1 antibody-based magnetic nanoparticles. Int J Nanomed 2273 (2013) 8:2273–84. doi: 10.2147/IJN.S45817 PMC3693816 23818783

[302] Wu S Liu X He J Wang H Luo Y Gong W. A dual targeting magnetic nanoparticle for human cancer detection. Nanoscale Res Lett (2019) 14:228. doi: 10.1186/s11671-019-3049-0 31289961 PMC6616609

[303] Krishna M Nadler SG. Immunogenicity to biotherapeutics – the role of anti-drug immune complexes. Front Immunol (2016) 7:21. doi: 10.3389/fimmu.2016.00021 26870037 PMC4735944

[304] Vaisman-Mentesh A Gutierrez-Gonzalez M DeKosky BJ Wine Y. The molecular mechanisms that underlie the immune biology of anti-drug antibody formation following treatment with monoclonal antibodies. Front Immunol (2020) 11:1951. doi: 10.3389/fimmu.2020.01951 33013848 PMC7461797

[305] Hansel TT Kropshofer H Singer T Mitchell JA George AJT. The safety and side effects of monoclonal antibodies. Nat Rev Drug Discovery (2010) 9:325–38. doi: 10.1038/nrd3003 20305665

[306] Wolbink GJ Aarden LA Dijkmans B. Dealing with immunogenicity of biologicals: assessment and clinical relevance. Curr Opin Rheumatol (2009) 21:211–5. doi: 10.1097/BOR.0b013e328329ed8b 19399992

