# Supplementary material for: Cell-targeted vaccines: implications for adaptive immunity
Source: Front Immunol. 2023 Aug 16;14:1221008. doi: 10.3389/fimmu.2023.1221008 (PMC10468591; doi:10.3389/fimmu.2023.1221008)
Supplement: Supplementary file 1 [file Table_1.docx]

Supplementary Material

Cell-targeted vaccines: Implications for adaptive immunity

**Trevor Ung^1^, Nakisha S. Rutledge^1^, Adam Weiss^1^, Aaron Esser-Kahn^1^, and Peter Deak^2^***

^1^Pritzker School of Molecular Engineering, University of Chicago, Chicago, IL 60637

^2^Chemical and Biological Engineering Department, Drexel University, Philadelphia, PA 19104

*** Correspondence:**

Center for Automation Technologies

3101 Ludlow St, Room 480

Philadelphia, PA 19104

[pd562@drexel.edu](mailto:pd562@drexel.edu)

215-895-6694

Supplementary Table 1: Summary of target cells and receptors, targeting methods, and outcomes.

| Target cell | Target receptor | Receptor Species | Targeting method | Vehicle | *In vivo*/  *In vitro* | Antigen | Adjuvant | Route of administration | CD8^+^ T cell response | CD4^+^ T cell response | Antibody response | Th bias | Reference |
| --- | --- | --- | --- | --- | --- | --- | --- | --- | --- | --- | --- | --- | --- |
| DC | CD11c | Mouse | αCD11c ScFv | DNA/recombinant protein | *In vivo* | HER2 | N/A | i.m. | Y | Y | Y | N/A | (*45*) |
| DC | CD11c | Mouse | αCD11c ScFv | Liposome | *In vivo* | OVA | IFN-γ, LPS | i.v. | Y | N/A | N/A | N/A | (*46*) |
| DC | CD11c | Mouse | ICAM-4 peptide | Polymersome | *In vivo* | N/A | N/A | i.v. | N/A | N/A | N/A | N/A | (*48*) |
| DC | N/A | Mouse | Peptide | Silica nanoparticle | *In vivo* | OVA | CpG | s.c. | Y | N/A | N/A | N/A | (*49*) |
| DC | N/A | Mouse | Peptide | Recombinant protein | *In vivo* | HPV | Flagellin | s.c. | Y | N/A | N/A | N/A | (*50*) |
| DC | CD11c | Mouse | αCD11c mAb | PLGA nanoparticle | *In vivo* | OVA | Poly(I:C)  R848 | s.c. | Y | N/A | N/A | N/A | (*51*) |
| DC | CD40 | Mouse | αCD40 mAb | PLGA nanoparticle | *In vivo* | OVA | Poly(I:C)  R848 | s.c. | Y | N/A | N/A | N/A | (*51*) |
| DC | CD11c | Mouse | αCD11c Fab | Conjugated protein | *In vivo* | OVA | αCD40 | i.v., s.c. | Y | Y | N/A | N/A | (*52*) |
| DC | MHC II | Mouse | αMHC II Fab | Conjugated protein | *In vivo* | OVA | αCD40 | i.v., s.c. | Y | Y | N/A | N/A | (*52*) |
| DC | CD11c | Mouse | αCD11c mAb | mAb/immune complex | *In vivo* | Goat IgG | N/A | i.d. | N/A | N/A | Y | N/A | (*53*) |
| DC | CD11c | Mouse | αCD11c mAb | mAb | *In vivo* | Rat IgG | N/A | s.c. | N/A | N/A | Y | N/A | (*55*) |
| DC | CD11c | Mouse | αCD11c ScFv | DNA/recombinant protein | *In vivo* | HA | N/A | i.m. | N/A | N/A | Y | N/A | (*56*) |
| DC | CD40 | Mouse | αCD40 ScFv | DNA/recombinant protein | *In vivo* | HA | N/A | i.m. | N/A | N/A | Y | Th1 | (*56*) |
| DC | MHC II | Mouse | αMHC II ScFv | DNA/recombinant protein | *In vivo* | HA | N/A | i.m. | N/A | N/A | Y | N/A | (*56*) |
| DC | CD11c | Mouse | αCD11c Fab | Conjugated protein | *In vivo* | OVA | CFA | i.v., s.c. | N/A | N/A | Y | N/A | (*57*) |
| DC | CD40 | Mouse | αCD40 Fab | Conjugated protein | *In vivo* | OVA | CFA | i.v., s.c. | N/A | N/A | Y | N/A | (*57*) |
| DC | MHC II | Mouse | αMHC II Fab | Conjugated protein | *In vivo* | OVA | CFA | i.v., s.c. | N/A | N/A | Y | N/A | (*57*) |
| DC | FLT3 | Mouse | FLT3L | DNA/recombinant protein | *In vivo* | HA | N/A | i.m. | N/A | N/A | Y | Th1 | (*56*) |
| DC | CD206 | Mouse | Mannose | Co-polymer | *In vivo* | OVA,  CSP | TLR7 agonist | i.d. | Y | Y | Y | N/A | (*64*) |
| DC | CD206 | Mouse | αCD206 mAb | Recombinant protein | *In vivo* | Hamster IgG, OVA | CpG, Ribi, | i.p. | Y | Y | Y | Th1 | (*66*) |
| DC | CD206 | Mouse | Sulfated glycans | Conjugated protein | *In vitro* | OVA | N/A | N/A | Y | Y | N | Th1 | (*65*) |
| cDC1 | XCR1 | Mouse | XCL1 | DNA/recombinant protein | *In vivo* | HA | N/A | i.m. | N/A | N/A | Y | Th1 | (*56*) |
| cDC1 | XCR1 | Mouse | XCL1 | DNA/recombinant protein | *In vivo* | OVA, HA | N/A | i.d. | Y | Y | Y | Th1 | (*93*) |
| cDC1 | XCR1 | Mouse | XCL1 | DNA/recombinant protein | *In vivo* | HA | N/A | i.d. | Y | Y | Y | Th1 | (*99*) |
| cDC1 | XCR1 | Mouse | XCL1 | DNA/recombinant protein | *In vivo* | OVA | N/A | i.d. | Y | Y | Y | Th1 | (*100*) |
| cDC1 | DEC-205 | Mouse | αDEC-205 mAb | Conjugated protein | *In vivo* | OVA | αCD40 | i.p., s.c. | Y | Y | N | Th1 | (*88*) |
| cDC1 | DEC-205 | Mouse | αDEC-205 ScFv | DNA/recombinant protein | *In vivo* | OVA | N/A | i.d. | Y | Y | Y | N/A | (*93*) |
| cDC1 | DEC-205 | Mouse | αDEC-205 mAb | Recombinant protein | *In vivo* | OVA | CpG | i.v. | Y | Y | Y | N/A | (*95*) |
| cDC1 | DEC-205 | Mouse | αDEC-205 ScFv | Recombinant protein | *In vivo* | OVA | αCD40 | s.c. | Y | N/A | N/A | N/A | (*98*) |
| cDC1 | DEC-205 | Human | αDEC-205 mAb | Recombinant protein | *In vitro* | NY-ESO-1 | N/A | N/A | Y | Y | N/A | Th1 | (*108*) |
| cDC1 | DEC-205 | Human | αDEC-205 mAb | Recombinant protein | *In vivo* | EBNA1 | Poly(I:C) | N/A | Y | Y | N/A | Th1 | (*109*) |
| cDC1 | DEC-205 | Human | αDEC-205 mAb | Recombinant protein | *In vivo* | NY-ESO-1 | Poly(I:C), R848 | i.d., s.c. | Y | Y | Y | Th1 | (*110*) |
| cDC1 | DEC-205 | Mouse | αDEC-205 mAb | Conjugated protein | *In vivo* | OVA | αCD40 | s.c. | Y | Y | N | Th1 | (*94*) |
| cDC1 | Clec12a | Mouse | αClec12a mAb | Recombinant protein | *In vivo* | OVA | CpG | i.v. | Y | Y | Y | N/A | (*95*) |
| cDC1 | Clec12a | Mouse | αClec12a mAb | Conjugated protein | *In vivo* | OVA | αCD40, CPG, LPS, Poly(I:C), | i.v. | Y | Y | Y | N/A | (*97*) |
| cDC1 | Clec9a | Mouse | αClec9a ScFv | DNA/recombinant protein | *In vivo* | OVA | N/A | i.d. | Y | Y | Y | Th1 | (*93*) |
| cDC1 | Clec9a | Mouse,  Human | αClec9a mAb | mAb | *In vivo* | Rat IgG | Alum, Poly(I:C) | i.v., s.c. | N/A | N/A | Y | N/A | (*114*) |
| cDC1 | Clec9a | Mouse | αClec9a mAb | Conjugated protein | *In vivo* | OVA | Curdlan, Poly(I:C) | i.v., s.c. | Y | Y | Y | Th1, Th17 | (*115*) |
| cDC1 | Clec9a | Mouse | αClec9a mAb | Recombinant protein | *In vivo* | OVA | CpG | i.v. | Y | Y | Y | Tfh | (*95*) |
| cDC1 | Clec9a | Mouse | αClec9a mAb | Recombinant protein | *In vivo* | OVA, HSV-1 | N/A | i.v. | N | Y | Y | Tfh | (*116*) |
| cDC1 | Clec9a | Mouse | αClec9a mAb | Recombinant protein | *In vivo* | OVA | N/A | i.v. | N | Y | Y | Tfh | (*117*) |
| cDC1 | Clec9a | Human | αClec9a mAb | Recombinant protein | *In vitro, In vivo* | NY-ESO-1 | Poly(I:C), R848 | s.c. | Y | N/A | N/A | N/A | (*96*) |
| cDC1 | TREML4 | Mouse | αTREML4 mAb | Recombinant protein | *In vivo* | OVA, HER2, GAGp24 | αCD40, poly(I:C) | i.p. | Y | Y | N/A | Th1 | (*90*) |
| cDC1 | TREML4 | Mouse | αTREML4 mAb | Recombinant protein | *In vivo* | MOG | N/A | s.c. | N/A | Y | N/A | N/A | (*91*) |
| cDC1 | CD36 | Mouse | αCD36 ScFv | Recombinant protein | *In vivo* | OVA | αCD40 | s.c. | Y | N/A | N/A | N/A | (*98*) |
| cDC1 | LOX1 | Mouse, Human | αLOX1  mAb | Conjugated protein | *In vivo* | OVA | IFA | s.c. | Y | N/A | N/A | N/A | (*102*) |
| cDC1 | LOX1 | Mouse | Hsp-60 | Recombinant protein | *In vivo* | OVA | N/A | s.c. | Y | N/A | N/A | N/A | (*103*) |
| cDC1 | LOX1 | Mouse, Human | αLOX1  mAb | Recombinant protein | *In vitro, In vivo* | HA, PSA | N/A | i.d. | N/A | Y | N/A | Th1 | (*104*) |
| cDC1 | FcγRIIb | Mouse | αFcγRIIb mAb | Conjugated protein | *In vivo* | OVA | αCD40, Poly(I:C) | i.p. | Y | Y | N/A | N/A | (*105*) |
| cDC1 | FcγRIII | Mouse | αFcγRIII mAb | Conjugated protein | *In vivo* | OVA | αCD40, Poly(I:C) | i.p. | Y | Y | N/A | N/A | (*105*) |
| cDC1 | FcγRIV | Mouse | αFcγRIV mAb | Conjugated protein | *In vivo* | OVA | αCD40, Poly(I:C) | i.p. | Y | Y | N/A | N/A | (*105*) |
| cDC1 | FcγRII | Human | IgG Fc | mAb/immune complex | *In vitro* | HCMV | LPS, Poly(I:C) | N/A | Y | Y | N/A | N/A | (*101*) |
| cDC2 | DCIR2 | Mouse | αDCIR2  mAb | Recombinant protein | *In vivo* | OVA | αCD40, LPS | i.p., i.v. | Y | Y | N/A | N/A | (*125*) |
| cDC2 | DCIR2 | Mouse | αDCIR2  mAb | Conjugated  protein | *In vivo* | NP hapten | Alum, CpG, Flagellin, Poly(I:C), pU/UC, R848 | i.v. | N/A | Y | Y | Th1 | (*126*) |
| cDC2 | DCIR2 | Mouse | αDCIR2  mAb | Recombinant protein | *In vivo* | OVA | αCD40, Poly(I:C) | i.p. | Y | Y | Y | Th1 | (*128*) |
| cDC2 | DCIR2 | Mouse | αDCIR2  mAb | Recombinant protein | *In vitro* | OVA | LPS | N/A | Y | Y | N/A | N/A | (*130*) |
| cDC2 | DCIR | Human | αDCIR  mAb | Recombinant protein | *In vitro* | FluMP, MART-1, GAGp24 | αCD40, LPS, Poly(I:C), R848 | N/A | Y | N/A | N/A | N/A | (*139*) |
| cDC2 | GM-CSFR | Mouse | GM-CSF | DNA/recombinant protein | *In vivo* | HA | N/A | i.m. | N/A | N/A | Y | Th2 | (*56*) |
| cDC2 | CCR1, CCR3, CCR5 | Mouse | CCL3 | DNA/recombinant protein | *In vivo* | HA | N/A | i.d., i.m. | N/A | Y | Y | Th2 | (*129*) |
| cDC2 | TLR5 | Mouse | Flagellin | DNA/recombinant protein | *In vivo* | HA | N/A | i.m. | N/A | N/A | Y | N/A | (*56*) |
| cDC2 | Dectin-1 | Mouse | αDectin-1 mAb | Conjugated protein | *In vivo* | OVA | Poly(I:C) | i.v., s.c. | Y | Y | Y | N/A | (*131*) |
| cDC2 | Dectin-1 | Mouse | αDectin-1 mAb | mAb | *In vivo* | Rat IgG | N/A | s.c. | N/A | N/A | Y | N/A | (*55*) |
| cDC2 | Dectin-2 | Mouse | αDectin-2 mAb | Conjugated protein | *In vivo* | OVA | Poly(I:C) | i.v., s.c. | Y | N/A | N/A | N/A | (*132*) |
| CDC2 | Dectin-2 | Mouse | αDectin-2 mAb | mAb | *In vivo* | Rat IgG | N/A | s.c. | N/A | N/A | Y | N/A | (*55*) |
| cDC2 | DC-SIGN | Mouse | αDC-SIGN mAb | mAb | *In vivo* | Rat IgG | CpG | s.c. | N/A | N/A | Y | N/A | (*133*) |
| cDC2 | DC-SIGN | Mouse | αDC-SIGN mAb | Conjugated protein | *In vivo* | OVA | αCD40 | s.c. | Y | Y | Y | N/A | (*134*) |
| cDC2 | FIRE | Mouse | αFIRE mAb | mAb | *In vivo* | Rat IgG | CpG | s.c. | N/A | N/A | Y | N/A | (*133*) |
| cDC2 | MGL | Human | Glycosylated MUC-1 peptide | N/A | *In vitro* | N/A | R848 | N/A | N/A | N/A | N/A | N/A | (*153*) |
| cDC2 | MGL | Human/Mouse | Peptide | N/A | *In vitro/In vivo* | N/A | N/A | i.p. | N/A | N/A | N/A | N/A | (*155*) |
| pDC | pDC-TREM | Mouse | αpDC-TREM mAb | mAb | *In vivo* | Rat IgG | N/A | s.c. | N/A | N/A | Y | N/A | (*55*) |
| pDC | Siglec H | Mouse | αSiglec H mAb | Conjugated protein | *In vivo* | OVA | CpG | i.v. | Y | N/A | N/A | N/A | (*162*) |
| pDC | Siglec H | Mouse | αSiglec H mAb | Recombinant protein | *In vivo* | OVA | Poly(I:C) | i.p. | N/A | Y | N/A | N/A | (*163*) |
| pDC | BST2 | Mouse | αBST2 mAb | Recombinant protein | *In vivo* | OVA | CpG, Poly(I:C) | i.p. | Y | Y | Y | Th1 | (*164*) |
| pDC | DEC-205 | Human | αDEC-205 mAb | PLGA nanoparticle | *In vitro* | gp100, tetanus toxoid | R848 | N/A | Y | Y | N/A | Th1 | (*158*) |
| pDC | DCIR | Human | αDCIR mAb | PLGA nanoparticle | *In vitro* | gp100, tetanus toxoid | R848 | N/A | Y | Y | N/A | Th1 | (*158*) |
| pDC | DCIR | Human | αDCIR mAb | Conjugated protein | *In vitro* | KLH | CpG | N/A | Y | Y | N/A | N/A | (*159*) |
| pDC | BDCA-2 | Human | αBDCA-2 mAb | PLGA nanoparticle | *In vitro* | gp100, tetanus toxoid | R848 | N/A | Y | Y | N/A | Th1 | (*158*) |
| pDC | BDCA-2 | Human | αBDCA-2 mAb | mAb | *In vivo* | N/A | CpG | i.p. | N/A | N/A | N/A | N/A | (*160*) |
| pDC | FcγRII | Human | αFcγRII mAb | PLGA nanoparticle | *In vitro* | gp100, tetanus toxoid | R848 | N/A | Y | Y | N/A | Th1 | (*158*) |
| pDC | FcγRII | Human | αFcγRII mAb | Conjugated protein | *In vitro* | N/A | CpG | N/A | N/A | N/A | N/A | N/A | (*161*) |
| CD103^+^ cDC1 | CD103 | Mouse | αCD103 mAb | Conjugated protein | *In vivo* | OVA | αCD40, LPS, Poly(I:C) | i.p. | Y | Y | Y | N/A | (*173*) |
| LC | CD207 | Human | Mannose | Mannosylated peptides | *In vitro* | gp100 | N/A | N/A | Y | N/A | N/A | N/A | (*183*) |
| LC | CD207 | Human | αCD207 mAb | Recombinant protein | *In vitro* | EBNA1 | Poly(I:C) | N/A | Y | Y | N/A | N/A | (*184*) |
| Mo | CD11b | Mouse | αCD11b mAb | Silica nanoparticle | *In vivo* | N/A | N/A | i.v. | N/A | N/A | N/A | N/A | (*191*) |
| Mo | Ly6C | Mouse | αLy6C mAb | Lipid nanoparticle | *In vivo* | N/A | N/A | i.v. | N/A | N/A | N/A | N/A | (*192*) |
| MΦ | F4/80 | Mouse | αF4/80 Fab | PLA nanoparticle | *In vivo* | N/A | N/A | Oral | N/A | N/A | N/A | N/A | (*206*) |
| MΦ | N/A | Rat | Tuftsin peptide | Alginate nanoparticle | *In vivo* | N/A | N/A | i.p. | N/A | N/A | N/A | N/A | (*211*) |
| MΦ | CD44 | Mouse | Hyaluronic acid | PLA nanoparticle | *In vitro* | N/A | N/A | N/A | N/A | N/A | N/A | N/A | (*213*) |
| MΦ | CD44 | Mouse | Hyaluronic acid | PEI nanoparticle | *In vivo* | N/A | N/A | i.p. | N/A | N/A | N/A | N/A | (*214*) |
| MΦ | CD206 | Human | Mannose | PEI nanoparticle | *In vitro* | N/A | N/A | N/A | N/A | N/A | N/A | N/A | (*215*) |
| MΦ | CD206 | Mouse | Mannose | PLGA nanoparticle | *In vivo* | N/A | N/A | i.v. | N/A | N/A | N/A | N/A | (*216*) |
| MΦ | Dectin-1 | Mouse | β-1,3-D-glucan | β-1,3-D-glucan microparticle | *In vivo* | N/A | N/A | i.p. | N/A | N/A | N/A | N/A | (*217*) |
| MΦ | Dectin-1 | Mouse | β-1,3-D-glucan | β-1,3-D-glucan microparticle | *In vivo* | N/A | N/A | i.p., oral | N/A | N/A | N/A | N/A | (*218*) |
| M2 MΦ | N/A | Mouse | Peptide | Fusion peptide | *In vivo* | N/A | N/A | i.v. | N/A | N/A | N/A | N/A | (*210*) |
| M2 MΦ | N/A | Mouse | Peptide | Fusion peptide | *In vitro* | N/A | N/A | N/A | N/A | N/A | N/A | N/A | (*219*) |
| M2 MΦ | N/A | Mouse | Melittin | Fusion peptide | *In vivo* | N/A | N/A | i.p. | N/A | N/A | N/A | N/A | (*220*) |
| M2 MΦ | N/A | Mouse | Melittin | Fusion peptide | *In vivo* | N/A | N/A | i.p. | N/A | N/A | N/A | N/A | (*221*) |
| M2 MΦ | FRβ | Mouse | Folate | Liposome | *In vivo* | N/A | N/A | i.v. | N/A | N/A | N/A | N/A | (*223*) |
| M2 MΦ | FRβ | Mouse | Folate | Liposome | *In vivo* | N/A | N/A | i.v. | N/A | N/A | N/A | N/A | (*224*) |
| M2 MΦ | FRβ | Mouse | αFrβ ScFv | Recombinant protein | *In vivo* | N/A | N/A | i.tu. | N/A | N/A | N/A | N/A | (*225*) |
| CD169^+^ MΦ | CD169 | Mouse | αCD169 mAb | Conjugated protein | *In vivo* | OVA | αCD40, Poly(I:C) | i.v. | Y | Y | N/A | Th1 | (*230*) |
| CD169^+^ MΦ | CD169 | Mouse | GM3 | Liposome | *In vivo* | OVA | αCD40, Poly(I:C) | i.v., s.c. | Y | Y | N/A | Th1 | (*231*) |
| CD169^+^ MΦ | CD169 | Mouse | Sialylated glycan | Liposome | *In vivo* | OVA | LPS, TLR7 agonist | i.v. | Y | Y | N/A | N/A | (*233*) |
| NΦ | N/A | Mouse | N/A, passive | Albumin nanoparticle | *In vivo* | N/A | N/A | i.v. | N/A | N/A | N/A | N/A | (*243*) |
| NΦ | N/A | Mouse | N/A, passive | Albumin nanoparticle | *In vivo* | N/A | N/A | i.v. | N/A | N/A | N/A | N/A | (*244*) |
| NΦ | Ly6G/Ly6C | Mouse | αLy6G/Ly6C mAb | PLGA-PEG nanoparticle | *In vivo* | N/A | N/A | i.n. | N/A | N/A | N/A | N/A | (*245*) |
| NΦ | Ly6G,  Neutrophil elastase | Mouse | αLy6G Fab, α1-sntitrypsin | Liposome | *In vivo* | N/A | N/A | i.v. | N/A | N/A | N/A | N/A | (*246*) |
| NΦ | CD177 | Mouse | Peptide | PLGA nanoparticle, liposome | *In vivo* | N/A | N/A | i.v. | N/A | N/A | N/A | N/A | (*248*) |
| Eo | Siglec-8 | Human | αSiglec-8 mAb | mAb | *In vivo* | N/A | N/A | i.v. | N/A | N/A | N/A | N/A | (*254*) |
| Eo | Siglec-F, Siglec-8 | Mouse, Human | Sialylated glycan | Liposome | *In vitro, in vivo* | N/A | N/A | i.v. | N/A | N/A | N/A | N/A | (*255*) |
| Ba/MC | CD33 | Human | Sialylated glycan | Liposome | *In vitro* | N/A | N/A | N/A | N/A | N/A | N/A | N/A | (*257*) |
| Ba/MC | CD33 | Human | Sialylated glycan | Liposome | *In vivo* | OVA, TNP | N/A | i.v. | N/A | N/A | N/A | N/A | (*258*) |
| Ba/MC | CD203c | Human | αCD203c mAb | Gold nanoparticle | *In vitro* | N/A | N/A | N/A | N/A | N/A | N/A | N/A | (*259*) |
| Ba/MC | CD203c | Human | αCD203c mAb | Gold nanoparticle | *In vitro* | N/A | N/A | N/A | N/A | N/A | N/A | N/A | (*260*) |
| B cell | CD21/35 | Mouse | C3b | Iron nanoparticle | *In vivo* | OVA | CpG | i.v. | N/A | Y | Y | Th1 | (*268*) |
| B cell | CD21/35, FcγR | Rat | C3b, IgM | Liposome | *In vivo* | N/A | N/A | i.v. | N/A | N/A | N/A | N/A | (*269*) |
| B cell | CD21/35, FcγR | Rat | C3b, IgM | Liposome | *In vivo* | N/A | N/A | i.v. | N/A | N/A | N/A | N/A | (*270*) |
| B cell | CD21/35, FcγR | Mouse | C3b, IgM | Liposome | *In vivo* | OVA | α-galactosyl-ceramide | i.v. | Y | N/A | N/A | N/A | (*271*) |
| B cell | CD21/35, FcγR | Mouse | C3b, IgM | Liposome | *In vivo* | OVA | α-galactosyl-ceramide | i.v. | N/A | N/A | Y | N/A | (*272*) |
| B cell | HEL BCR | Mouse | HEL | CaP nanoparticle | *In vitro* | HEL | N/A | N/A | N/A | N/A | N/A | N/A | (*273*) |
| B cell | HEL BCR | Mouse | HEL | CaP nanoparticle | *In vivo* | HEL, OVA | CpG, Flagellin, Poly(I:C), R848, tetanus toxoid | i.m., i.v., s.c. | N/A | Y | Y | N/A | (*274*) |
| B cell | VRC01 BCR | Mouse | eOD-GT8 | Alum nanoparticle | *In vivo* | eOD-GT8, MD39 | Alum, Quil-A | s.c. | N/A | N/A | Y | N/A | (*275*) |
| B cell | IgM | Mouse | αIgM mAb | mAb/immune complex | *In vivo* | pigeon cytochrome c | N/A | i.v. | N/A | Y | N/A | N/A | (*278*) |
| B cell | CD19 | Mouse | αCD19 mAb | mAb/immune complex | *In vivo* | pigeon cytochrome c | N/A | i.v. | N/A | Y | N/A | N/A | (*278*) |
| B cell | CD19 | Mouse | αCD19 ScFv, αCD19 mAb | Recombinant protein, conjugated protein | *In vivo* | OVA, HER2 | N/A | i.v. | Y | Y | Y | Th2 | (*279*) |
| B cell | CD19 | Mouse | αCD19 mAb | Conjugated protein | *In vivo* | OVA, MUC1 | CpG | i.v. | Y | Y | Y | Th1 | (*280*) |
| B cell | MHC II | Mouse | αMHC II ScFv | DNA/recombinant protein | *In vivo* | OVA, HA | N/A | i.d., i.v. | N/A | Y | Y | Tfh | (*281*) |
| B cell | MHC II | Mouse | αMHC II ScFv | DNA/recombinant protein | *In vivo* | OVA, HA | N/A | i.d., i.m., i.v. | Y | Y | Y | N/A | (*282*) |
| FDC | CD21 | Mouse | C3b | Gold nanoparticle | *In vivo* | OVA | N/A | i.d. | N | N | Y | N/A | (*287*) |
| FDC | CD21 | Mouse | CTA1-DD | Recombinant protein | *In vivo* | OVA, NP hapten | CTA1-DD | i.n., i.p., i.v. | N/A | Y | Y | N/A | (*288*) |
| FDC | CD21 | Mouse | CTA1-DD | Recombinant protein | *In vivo* | OVA, NP hapten, M2e | CTA1-DD | i.n., i.v., s.c., oral | N/A | Y | Y | Tfh | (*289*) |
| FDC | CD35 | Mouse | αCD35 ScFv | Recombinant protein | *In vivo* | eOD-GT8 | saponin | s.c. | N/A | N/A | Y | N/A | (*290*) |
| LEC | LYVE-1 | Mouse | αLYVE-1 mAb | Iron nanoparticle | *In vitro* | N/A | N/A | N/A | N/A | N/A | N/A | N/A | (*301*) |
| LEC | LYVE-1, gp38 | Mouse | αLYVE-1 mAb, gp38 mAb | Iron nanoparticle | *In vivo* | N/A | N/A | i.v. | N/A | N/A | N/A | N/A | (*302*) |

Abbreviations: DC: dendritic cell; cDC1, conventional type 1 dendritic cell; cDC2: conventional type 2 dendritic cell; pDC: plasmacytoid dendritic cell; LC: Langerhans cell; Mo: monocyte; MΦ: macrophage; NΦ: neutrophil; Eo: eosinophil; Ba: basophil; MC: mast cell; FDC: follicular dendritic cell; LEC: lymphatic endothelial cell; mAb: monoclonal antibody; Fab: fragment antigen-binding; ScFv: single chain variable fragment; i.d.: intradermal; i.m.: intramuscular; i.n.: intranasal; i.p.: intraperitoneal; i.tu.: intratumoral; i.v.: intravenous; s.c.: subcutaneous
